# Mechanochemical Decoupling of ATP Hydrolysis and RNA Translocation in SARS-CoV‑2 nsp13 by the L405D Mutation

**DOI:** 10.1021/acs.jpcb.6c03935

**Published:** 2026-09-09

**Authors:** Elham Fazelpour, Priti Roy, Kole J. Frederick, Martin McCullagh

**Affiliations:** Department of Chemistry, 7618 Oklahoma State University, Stillwater, Oklahoma 74078, United States

## Abstract

SARS-CoV-2 nonstructural protein 13 (nsp13) is a highly conserved helicase that couples ATP hydrolysis to RNA translocation through long-range allosteric communication between its ATPase and RNA-binding domains. In prior work, we identified L405 as a key regulator of interdomain motions and proposed that the L405D mutation would disrupt this coupling by perturbing conformational transitions required for translocation [ J. Phys. Chem. B 2024, v128, 492–503 10.1021/acs.jpcb.3c06714PMC1125656338175211]. Subsequent experiments confirmed that L405D attenuates helicase activity and ATPase activity, implicating the potential formation of a new salt bridge, D405-R560, for both effects [ J. Biol. Chem. 2026, v302, 111198 10.1016/j.jbc.2026.111198PMC1293004941581884]. Here, we provide a data-driven explanation for both effects by combining Gaussian accelerated molecular dynamics (GaMD) simulations with Shape-GMM clustering and linear discriminant analysis. Whereas wild-type nsp13 exhibits both conformational selection and induction, L405D collapses the conformational landscape to operate predominantly through selection, with ATP-induced structural transitions observed in the wild-type ensemble no longer sampled by the mutant. This loss of induction likely impairs ATP turnover while simultaneously disrupting coordinated motif–RNA interactions required for inchworm translocation. These findings demonstrate how mutation-induced reshaping of conformational ensembles can modulate access to reaction-competent states, providing a general framework for understanding how targeted mutations disrupt catalytic function through allosteric ensemble remodeling in motor proteins.

## Graphical abstract

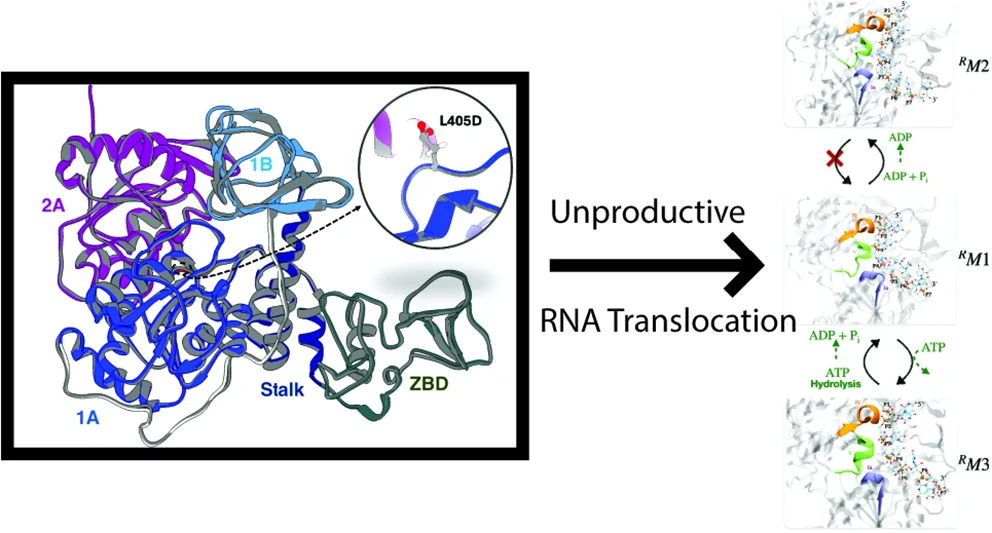

## Introduction

The SARS-CoV-2 nonstructural protein 13 (nsp13) helicase is an essential viral motor whose function depends on coupling ATP hydrolysis to directional RNA translocation during genome replication. Like other superfamily 1 (SF1) helicases,^1,2^ nsp13 uses the free energy of nucleotide hydrolysis to unwind RNA duplexes and translocate along single-stranded RNA, enabling replication and transcription of the viral genome.^3–5^ Because disruption of this mechanochemical coupling can stall viral replication without necessarily preventing nucleotide binding or hydrolysis, nsp13 has emerged as an attractive target for antiviral drug development.^6,7^ Structural studies have shown that nsp13 contains two RecA-like ATPase domains (1A and 2A), an RNA-binding 1B domain, a stalk domain, and an N-terminal zinc-binding domain (ZBD), with long-range communication between the ATP-binding pocket and the RNA-binding cleft governing productive translocation (Figure 1).^8–12^ Understanding how this allosteric coupling is regulated is therefore central to explaining helicase function and dysfunction.

**Figure 1:**
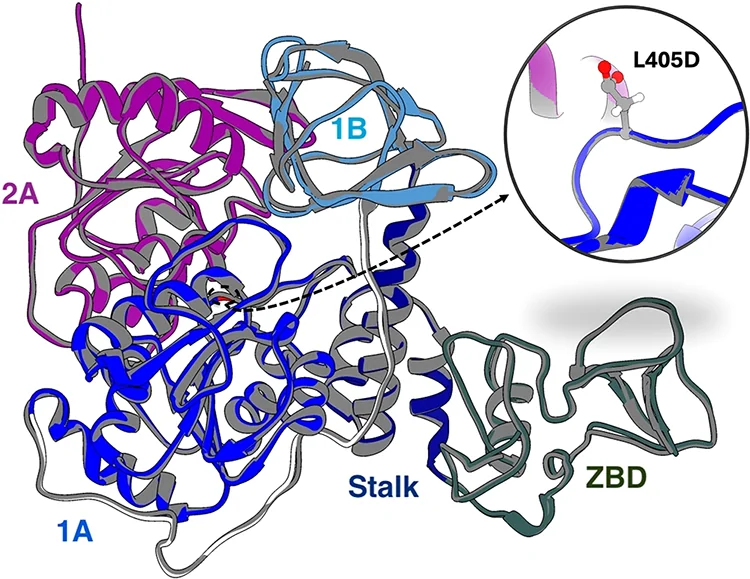
Depiction of SARS-CoV-2 nsp13 structure and substrate binding sites. Overlay of the L405D mutated nsp13 structure depicted with distinct subdomain colors and the SARS-CoV-2 structure (PDB: 7NN0 ^13^) in gray.

ATP-driven RNA translocation in helicases is governed by shifts in conformational ensembles, where ATP binding both selects pre-existing states and induces new reaction-competent conformations.^14–16^ In this framework, functional transitions arise through a balance between conformational selection and conformational induction that regulates interdomain cleft opening, motif–RNA contacts, product release, and directional stepping.^16–22^ Although nsp13 belongs to the SF1 helicase superfamily rather than SF2,^10^ it employs a related inchworm-like translocation mechanism in which coordinated binding and release of RNA by motifs Ia, IV, and V drive one-base movement along single-stranded RNA.^15,23–26^ Productive ATP hydrolysis therefore requires precise allosteric communication between the ATP-binding pocket formed by the 1A and 2A domains and the RNA-binding cleft formed by the 1A, 1B, and 2A domains. Mutations that perturb this communication can preserve ATP binding while disrupting mechanical translocation, leading to stalled or non-productive helicase cycles.

Previous computational and experimental studies identified L405 as a key allosteric regulator of ATP-dependent conformational transitions in nsp13.^16,27^ In our prior work, simulations along the ATP hydrolysis cycle showed that the presence of ATP does not substantially reorganize the ATP pocket itself, but instead shifts the relative positioning of the 1A, 1B, and 2A domains and alters the probability of RNA-cleft states associated with inchworm translocation.^14,16^ Linear discriminant analysis of these conformational changes identified residue L405, highlighted in the inset of Figure 1, as the strongest discriminator of open and closed interdomain states, indicating that it plays a central role in organizing the conformational ensemble required for productive ATP-driven translocation. Cryptic pocket analysis using PocketMiner^28^ further revealed that L405 is embedded within a transient, state-dependent pocket that emerges during the catalytic cycle (Figure S1), suggesting a regulatory role in stabilizing ATP-dependent structural transitions.^16^ Guided by these observations, we proposed the L405D mutation as a means to disrupt ATP-to-RNA coupling. Subsequent experiments confirmed that L405D attenuates helicase activity while also reducing ATPase function, both of which are attributed to the mutant aspartate potentially competing with D534 for a salt-bridge with R560.^27^

The central unresolved question is whether the L405D mutation disrupts only the ATP pocket or also the communication between the ATP pocket and RNA-cleft. We hypothesize that L405D reshapes the conformational ensemble of nsp13 by suppressing ATP-induced conformational induction, such that the enzyme operates predominantly through conformational selection. Specifically, we propose that the mutation replaces the directional four-state wild-type progression with a restricted three-state ensemble that fails to couple nucleotide turnover to productive domain rearrangement. At the molecular level, we further propose that this conformational reshaping originates from perturbation of the native D534–R560 electrostatic network within Motif V, which may disrupt communication between the ATPase site and the RNA-binding cleft, thereby perturbing the coordinated motif–RNA register shifts associated with productive inchworm translocation. To test this hypothesis, we performed Gaussian accelerated molecular dynamics (GaMD) simulations across four hydrolysis states (ssRNA, ssRNA+ATP, ssRNA+ADP+P_*i*_, and ssRNA+ADP), followed by Shape-GMM clustering and linear discriminant analysis to characterize mutation-dependent changes in protein and RNA-cleft conformational ensembles. These results provide a molecular mechanism for how mutation-induced ensemble remodeling decouples chemical and mechanical function in a viral motor protein.

## Methods

### Starting Structure

To generate the mutated SARS-CoV-2 nsp13 helicase structure from the wild-type, we used crystallographic data (PDB: 7NN0 ^13^), with a resolution of 3.04 Å. This structure was co-crystallized with several components, including the substrate ANP, magnesium as a cofactor, water molecules, and zinc ions within the ZBD. Additionally, ssRNA was incorporated from an aligned RNA-bound Upf1 helicase structure (PDB: 2XZL^29^) as described in our prior study.^14^ We introduced the mutation by substituting LEU405 with ASP405, and applied this modification consistently across the substrate-bound states: ssRNA, ssRNA+ATP, ssRNA+ADP+P_*i*_, and ssRNA+ADP, in line with the wild-type simulation protocol.^16^

### System Preparation

For nsp13 and ssRNA, the force field parameters used were ff14SB^30^ and ff99bsc0*χ*OL3,^29,31^ respectively. ATP and ADP parameters were sourced from the AMBER parameter database.^32^ The P_*i*_ parameter was taken from previous work by our group,^33^ and the three zinc ions in the nsp13 ZBD were parametrized using the MCPB module in AMBER.^34^ These zinc ions bond with residues in the following arrangements: Cys-Cys-Cys-Cys, Cys-Cys-Cys-HID, and Cys-Cys-HID-HIE in their respective pockets. Each substrate state was solvated using the TIP3P water model with at least a 12 Å buffer, resulting approximately 207 K atoms after adding Na^+^ and Cl^–^ ions to neutralize the charge and mimic a 0.1 M salt concentration.

### Simulation Details

All systems underwent energy minimization with a gradual removal of solute position restraints across 10 stages, each involving 2000 steps of steepest descent minimization. In the initial minimization step, a restraint of 500 kcal·mol^–1^·Å^–2^ was applied to all atoms except water (excluding crystal water) and salt. The restraint was then progressively reduced to 50.0, 10.0, 1.0, and 0.1 kcal·mol^–1^·Å^–2^ on the protein side chains, RNA, substrate, and Mg^2+^ ion over four steps. Subsequently, restraints on the protein backbone were gradually lowered in four phases, using 50.0, 10.0, 1.0, and 0.1 kcal·mol^–1^·Å^–2^. The final minimization run was conducted without any restraints. Following minimization, protein complexes were heated incrementally from 0 to 300 K over 1 ns with a harmonic restraint of 40 kcal·mol^–1^·Å^–2^ on protein, RNA, and substrate atoms. Following heating, six stages of pressure equilibration to 1 atm were conducted with progressively reduced harmonic restraints of 40.0, 20.0, 10.0, 5.0, 1.0, and 0.1 kcal·mol^–1^·Å^–2^ on all protein and ligand atoms. The first equilibration stage was run for 1 ns, while the subsequent stages each ran for 200 ps. A restraint-free production run was then performed at constant temperature and pressure (*NPT*) for 10 ns, repeated in triplicate. In the ssRNA + ADP + P_*i*_ substrate state, this step was extended to 500 ns to ensure that the Mg^2+^ ion’s octahedral coordination was conserved, particularly with SER289.^35^ From this production phase of conventional molecular dynamics (cMD), we recorded the minimum, average, maximum, and standard deviation values of the system’s potential energy. After the initial cMD simulations, we utilized Gaussian accelerated molecular dynamics (GaMD) simulations to improve conformational sampling across all systems. This approach included applying a boost to the entire system and dihedral potentials (igamd = 3), while keeping the other GaMD parameters at their default settings in Amber 18 ^36^ (σ_0*P*_ = 6 kcal·mol^–1^, σ_0*D*_ = 6 kcal·mol^–1^, *iE* = 1 or *E* = *V*_max_, nteb = 20,000,000, and ntave = 1,000,000 steps). Following the protocol, GaMD simulations commenced from equilibrated states obtained through brief cMD runs. All simulations were conducted using the GPU-accelerated AMBER18 package^36^ with a 2 fs integration time step, while hydrogen atoms were constrained with the SHAKE algorithm.^37^ Long-range electrostatic interactions were calculated using Particle Mesh Ewald,^38^ applying a nonbonded interaction cutoff of 12 Å. Pressure and temperature were controlled using a Monte Carlo barostat and a Langevin thermostat, respectively. We conducted at least three GaMD simulations for each system, resulting in around 9 μs per substrate state. In total, the ensembles accumulated to approximately 36 *μ*s. A summary of performed simulations is provided in Table S1 in the supporting information.

### Shape-GMM Clustering

To quantify mutation-dependent changes in the conformational ensemble of nsp13 across the ATP hydrolysis cycle, we employed the size-and-shape space Gaussian mixture model (Shape-GMM) clustering algorithm.^39–41^ Shape-GMM provides a rigorous structural clustering framework that operates directly in aligned Cartesian coordinate space, allowing conformational states to be identified from collective domain motions without requiring predefined reaction coordinates or low-dimensional projections. This is particularly important for ATP-driven molecular motors such as nsp13, where function emerges from coordinated interdomain rearrangements rather than single geometric observables.

To choose an appropriate number of components/clusters for each feature choice we use the elbow heuristic coupled with cross validation as described in the shapeGMM literature.^39–41^ That is, we scan the number of components and choose the number of components that minimizes the second derivative of the log-likelihood with respect to the number of components. See Figures S3a and S4 for examples.

The effect of sampling on cluster populations is estimated by fitting 30 shapeGMM objects using different randomly selected training sets. Each training set has equal weight from each substrate state. Following clustering, the cluster probabilities are reweighted for each replica in each hydrolysis state by using a second-order cumulant expansion of the boost potential.^42^ Errors are reported as the standard error from the three replicas. A summary of number of frames used in Shape-GMM clustering of RNA-cleft and protein is provided in Table S1.

### Linear Discriminant Analysis

Linear discriminant analysis (LDA) was used to identify the structural coordinates that most strongly distinguish functionally relevant conformational states and to define low-dimensional reaction coordinates for ATP-dependent transitions in nsp13. Unlike unsupervised dimensionality reduction methods, LDA is a supervised approach that maximizes variance between predefined conformational clusters while minimizing variance within each cluster, making it particularly well suited for identifying the dominant collective motions associated with ATP hydrolysis, product release, and RNA translocation.

Using cluster assignments obtained from Shape-GMM, aligned atomic coordinates were projected onto C–1 discriminant vectors (where C is the number of conformational clusters) that optimally separate the states.^43^ These discriminant vectors provide an interpretable approximation to the reaction coordinate by highlighting the domain motions and residue-level displacements most strongly associated with transitions between clusters. In particular, the magnitude of individual residue contributions along the discriminant vectors was used to identify residues that act as allosteric regulators of conformational change. In our previous work, this analysis identified L405 as the strongest contributor to ATP-dependent opening and closing motions between the 1A, 1B, and 2A domains, motivating its selection for mutational analysis in the present study.

LDA was performed using the scikit-learn^44^ Python library with the singular value decomposition solver to ensure numerical stability in the high-dimensional coordinate space. The resulting discriminant projections were used both to assess cluster separability and to characterize the relationship between local residue perturbations and global conformational transitions.

### Model Corroboration

#### Presence of SF1 Helicase-like Conserved Contacts in the ATP Pocket and the RNA Cleft

In our current models, the ssRNA is derived from the crystal structure of the Upf1 helicase, while all substrates are produced through slight modifications of ANP, which is co-crystallized with SARS-CoV-2 nsp13.^13^ Given these modifications, it is crucial to assess the stability of these insertions by comparing protein contacts with those observed in crystal structures of other SF1 helicases. The contact between the SARS-CoV-2 nsp13 protein and its bound ligands including ssRNA, ssRNA+ATP, ssRNA+ADP+P_*i*_, and ssRNA+ADP systems are compared with analogous contacts observed in other SF1 helicases, such as Upf1 ^29^ and IGHBMP,^45^ to demonstrate that the selected starting structures are appropriate and remain stable during the simulations. A contact is considered to occur when a protein residue lies within 5 Å of ATP, ADP + P_*i*_, or ADP. The identities of these residues, along with the likelihood of their interactions with the ligands, are shown in Table S1, focusing on residues from key functional motifs.

#### Stability of Mg^2+^ Coordination Geometry Throughout the Simulations

As an additional measure of active-site fidelity, we monitored the distance between the catalytic Mg^2+^ ion and the SER289 side-chain hydroxyl oxygen (OG) throughout the GaMD production trajectories of the ssRNA+ATP, ssRNA+ADP+P_*i*_, and ssRNA+ADP systems (*n* = 3 replicates per state; Figure S2). Across all replicates and nucleotide states, the Mg^2+^–SER289 distance remains stable at 2.0–2.5 Å on average, consistent with canonical, well-maintained octahedral Mg^2+^ coordination geometry.^13,46^ This stability indicates that the modeled active-site geometry is preserved throughout the simulations and supports the structural fidelity of the starting systems used for downstream mechanistic interpretation.

## Results and Discussion

We propose that the experimentally observed attenuation of ATPase and helicase activities in L405D arises from altered ATP-dependent conformational sampling and increased population of non-productive conformational states. In wild-type helicase function, the inchworm stepping cycle couples ATP turnover to directional ssRNA movement through coordinated communication between the ATP-binding pocket and the RNA-binding cleft, such that ATP binding, hydrolysis, and product release drive synchronized motif–RNA interactions required for forward translocation.^27^ We hypothesize that replacing L405 with aspartate disrupts this mechanochemical coupling by reshaping the conformational ensemble of nsp13, restricting access to reaction-competent states across the hydrolysis cycle, which we hypothesize may reduce the efficiency of product release. To test this model, we analyze four hydrolysis-state systems (ssRNA, ssRNA+ATP, ssRNA+ADP+P_*i*_, and ssRNA+ADP) using Gaussian accelerated molecular dynamics simulations followed by Shape-GMM clustering and linear discriminant analysis. By comparing mutant conformational clusters to the corresponding wild-type ensembles, we distinguish whether ATP-dependent transitions arise through conformational induction (newly accessed states) or conformational selection (redistribution among pre-existing states). We first examine how L405D disrupts the motif Ia/IV/V interactions required for inchworm RNA translocation, then examine how the mutation alters ATP-dependent conformational induction and identify changes in conformational sampling that are consistent with reduced efficiency of product release. Together, these analyses provide a molecular mechanism for how a single allosteric mutation decouples ATPase chemistry from productive mechanical translocation.

Throughout the following discussion, conformational clusters are labeled according to the feature set and protein variant: R denotes RNA-cleft clusters and P denotes full-protein conformational clusters, while M and W denote the L405D mutant and wild type, respectively. Thus, ^R^M1–^R^M4 and ^R^W1–^R^W3 refer to mutant and wild-type RNA-cleft clusters, whereas ^P^M1–^P^M3 and ^P^W1–^P^W4 refer to mutant and wild-type protein conformational clusters.

### L405D Mutation Disrupts the Inchworm Mechanism of RNA Translocation

L405D disrupts the coordinated motif–RNA interactions required for directional inchworm translocation in nsp13. In the wild-type helicase, three states of RNA were identified (Figure 2b).^13,14,16^ Characterizing these three states and their ATP-dependence led to the description that motifs Ia, IV, and V within the RNA-binding cleft execute synchronized cycles of loose-to-tight RNA binding that maintain a characteristic two-phosphate separation between the leading contact (motif IV) and trailing contacts (motifs Ia and V), enabling one-base (5′ → 3′) stepping per ATP hydrolysis cycle.^13,14,16^ This coordinated inchworm mechanism couples ATP turnover to productive RNA unwinding during viral replication.^47,48^ Because L405 is positioned adjacent to motif V at a critical allosteric hinge, the L405D mutation is expected to perturb both RNA geometry and communication between the ATP-binding pocket and the RNA-binding motifs. Rather than supporting a single ATP-synchronized translocation pathway, the mutant is hypothesized to populate persistent, non-productive RNA-binding states that may disrupt coordinated stepping.^27^

**Figure 2:**
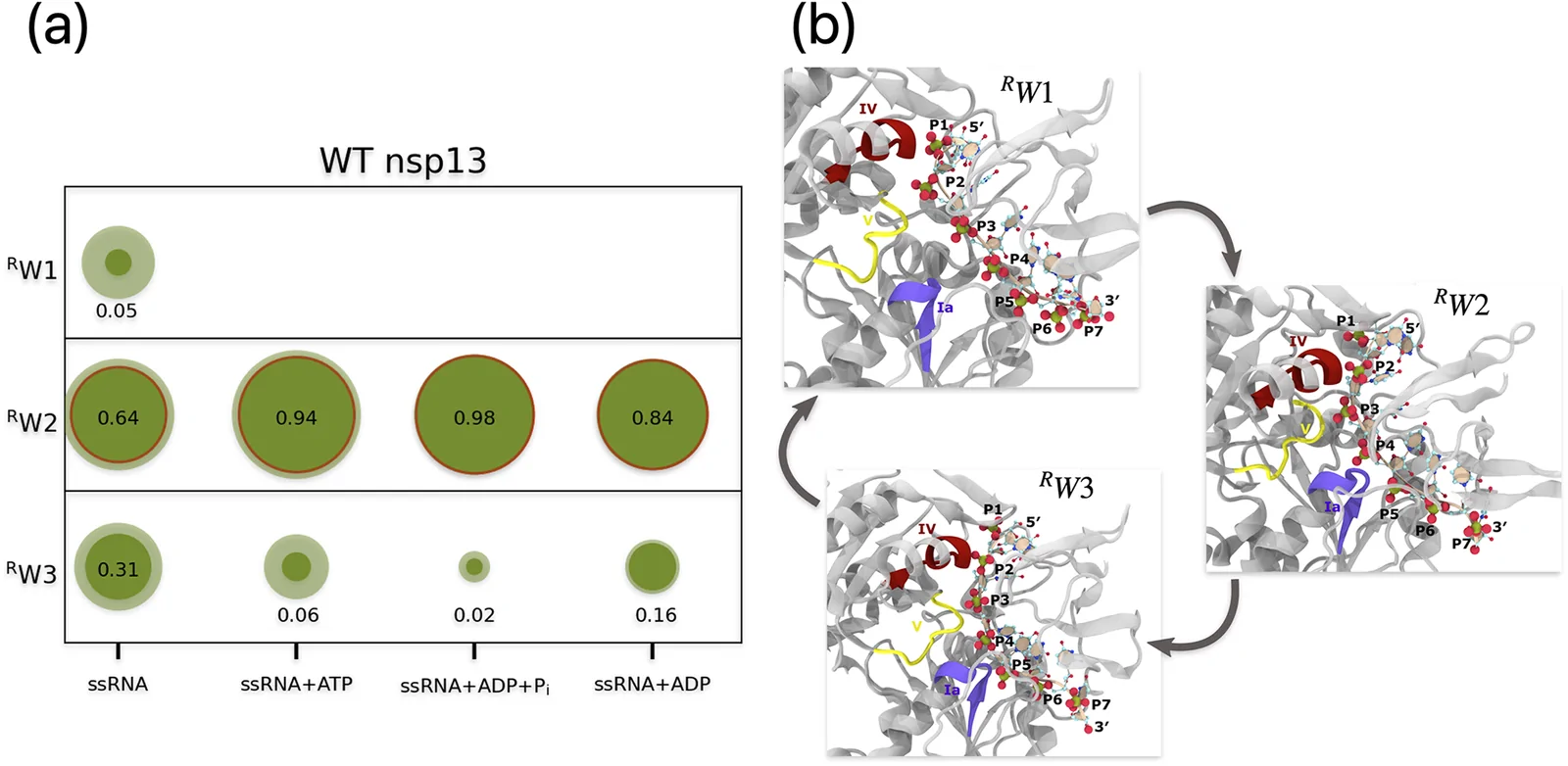
(a) Reweighted populations of RNA cleft clusters in wild type (WT) nsp13 denoted ^R^W1–^R^W3, presented in scatter form, where the size of each filled circle is proportional to the respective cluster’s probability and the surrounding halo represents the associated uncertainty. (b) Structural images represent RNA cleft conformations (^R^Ws) identified through weighted shape-GMM clustering of motifs Ia, IV, and V and ssRNA phosphate positions. The proposed ^R^W1 →^R^W2 →^R^W3 →^R^W1 cycle involves sequential rearrangement of motif–phosphate contacts, resulting in the forward movement of nsp13 by one phosphate toward the 3′end of the RNA.^15^ Arrows indicate the proposed sequence of conformational transitions.

L405D expands the RNA ensemble into four distinct conformational states, including non-productive binding configurations absent in wild-type nsp13. To characterize these cleft rearrangements, clustering was performed using the phosphate atoms of the bound ssRNA, with trajectories from all hydrolysis states combined prior to analysis. The optimal number of RNA conformational clusters (^R^M1–^R^M4) was determined to be four based on the plateau in log-likelihood improvement and the minimum in the second derivative of the training-set curve (Figure S2a). Linear discriminant analysis further showed clear separation among these clusters (Figure S2b): LD1 primarily distinguishes the apo-like ^R^M1 from the more advanced states ^R^M2 and ^R^M4, while LD2 and LD3 resolve the distinct positioning of ^R^M3 and ^R^M4. Replica specific cluster populations are provided in Table S2 while values averaged over replicas are provide in Figure 3a. Compared to the three-state RNA-cleft ensemble observed in wild-type nsp13, the mutant therefore samples a broader and less coordinated conformational landscape, indicating that L405D introduces additional substates that compete with productive ATP-coupled translocation.

**Figure 3:**
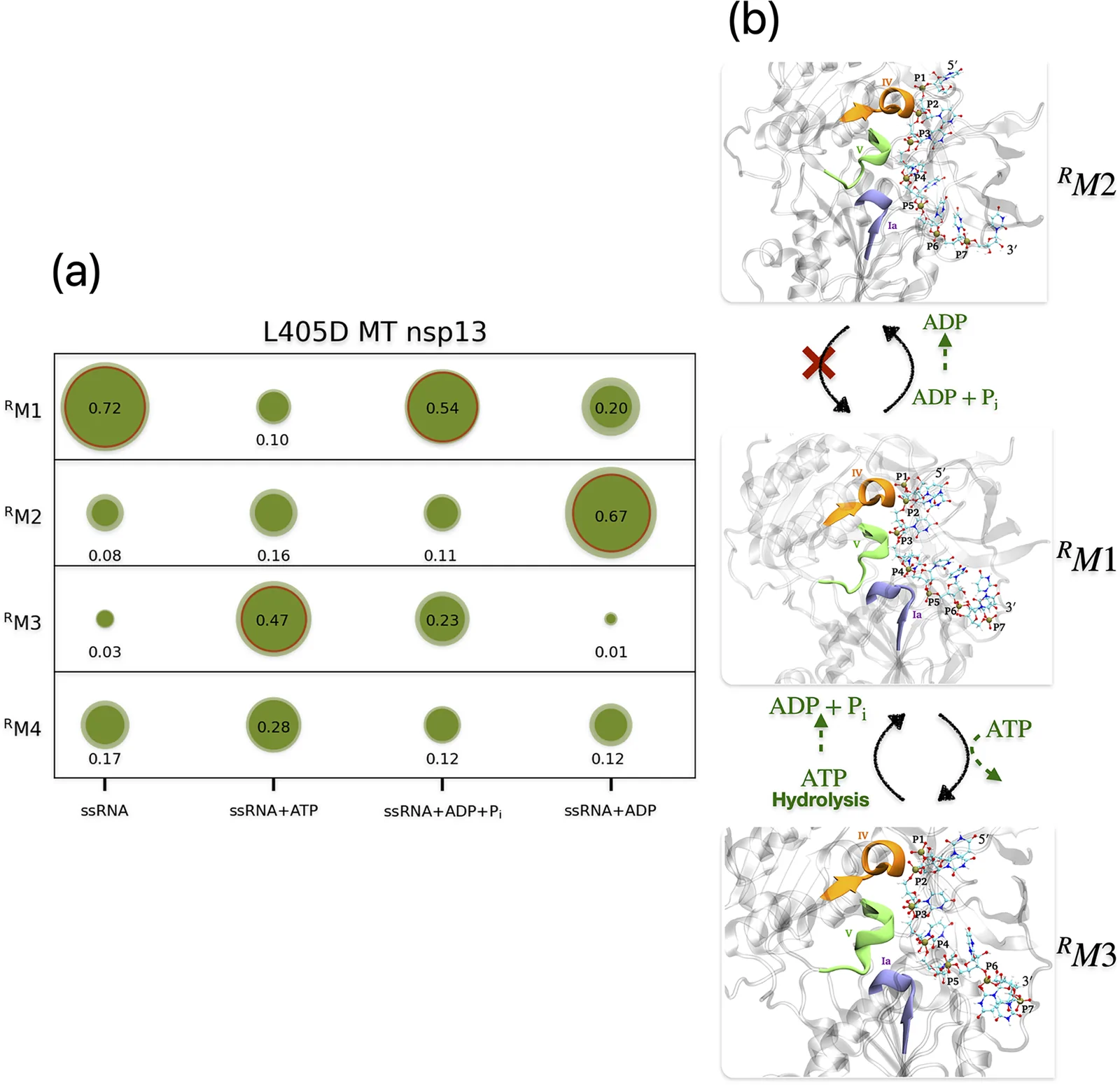
RNA clustering and proposed disruption of directed translocation in the L405D mutant (MT) of nsp13. Clusters are denoted ^R^M1–^R^M4 and determined from Shape-GMM clustering on the C_*α*_ positions of residues in motifs Ia, IV, and V and ssRNA phosphates. (a) Reweighted probability of RNA cleft clusters, presented in scatter form, where the size of each filled circle is proportional to the respective cluster’s probability and the surrounding halo represents the standard error from the three replicas. (b) Representative structures of each RNA cleft cluster identified by the frame closest to the mean. Arrows indicate the proposed ^R^M1 →^R^M3 →^R^M1 →^R^M2 sequence imposed by L405D, in contrast to the productive wild type (WT) translocation cycle proposed in Figure 2b. ATP-driven advancement from ^R^M1 to ^R^M3 is followed by a reversal upon hydrolysis rather than progression toward the next translocation state. The strong population of the tight-grip RM2 conformation in the ADP-bound ensemble may be unfavorable for ADP release.

ATP turnover no longer drives a single productive RNA-cleft progression in the L405D mutant, but instead redistributes the enzyme among competing non-productive substates. In the ssRNA-bound state, the ensemble is dominated by the apo-like ^R^M1 cluster (0.72 ± 0.18), with only minor populations of the remaining clusters, Figure 3a, establishing ^R^M1 as the principal nucleotide-free conformation. ATP binding redistributes the population across all four clusters and shifts the dominant population to ^R^M3 (0.47 ± 0.18), with secondary occupancy of ^R^M4 (0.28 ± 0.07) and ^R^M2 (0.16 ± 0.10), indicating that nucleotide binding drives a partial register shift rather than stabilizing a unique translocation-ready state. In the ADP+P_*i*_ state, the ensemble returns to ^R^M1 (0.54 ± 0.20) as the dominant cluster, with partial occupancy of ^R^M3 (0.23 ± 0.11) and ^R^M4 (0.12 ± 0.04), indicating that hydrolysis reverses rather than advances the RNA-cleft progression. Following the phosphate release, the ADP-bound state shifts toward ^R^M2 (0.67 ± 0.29) and ^R^M1 (0.20 ± 0.19), rather than progressing to a distinct post-hydrolysis conformation poised for reset. Unlike wild-type nsp13, where ATP binding and hydrolysis drive a coordinated and directional progression of RNA-cleft states, the L405D mutant repeatedly revisits overlapping conformations without establishing a single forward-translocation pathway. This loss of ATP-synchronized cleft progression provides a structural basis for how nucleotide turnover may become decoupled from productive RNA movement. We note that the absolute cluster populations show substantial variability across individual GaMD replicas (Table S2), consistent with individual trajectories sampling distinct metastable basins. Accordingly, we focus our mechanistic interpretation primarily on qualitative substrate-dependent population shifts that are reproduced across the ensemble of independent simulations rather than on the precise absolute populations of individual clusters.

L405D disrupts the canonical two-phosphate separation required for directional inchworm stepping. In productive wild-type translocation, motif IV serves as the leading RNA contact while motifs Ia and V act as trailing contacts, maintaining a characteristic two-phosphate gap that enables one-base forward movement during each ATP hydrolysis cycle.^16^ As shown in Tables 1 and 2, which quantify the motif–RNA contacts across all four conformational clusters of the L405D mutant, this coordination is systematically disrupted. Motif IV remains tightly associated with phosphate P2 across nearly all clusters, but the positioning of motifs Ia and V becomes uncoupled from this register, the precise motif-to-phosphate alignment required for directional stepping. In ^R^M1, the dominant ssRNA and ADP+P_*i*_ state, motif IV contacts P2 tightly (5.5(1) Å), while motifs Ia and V remain relatively distant (7.0(1) and 6.6(6) Å, respectively), with motif Ia predominantly contacting P5 (63%), consistent with a disengaged pre-translocation state. Critically, the return to RM1 upon hydrolysis, rather than progression to a new register, is inconsistent with the directional conformational progression proposed for wild type. The transition to ^R^M3, the dominant ATP-bound state, yields a close contact between RNA and motifs Ia and V (6.5(1) and 5.9 (5) Å) while motif IV loosens (6.6(9) Å). In ^R^M2, the dominant ADP-bound state, all three motifs simultaneously adopt tight RNA contacts across the entire cycle (5.3(1)–6.0(6) Å), with motif Ia re-engaging P4 (99.7%). This configuration may represent a non-productive state, in which the enzyme maintains tight RNA contacts while bound to ADP, a geometry that may be unfavorable for RNA release and ADP dissociation during productive reset. Critically, the canonical two-phosphate gap between motif IV and Ia is not preserved, and no cluster supports the forward power-stroke register seen in wild-type.

**Table 1:** Weighted Average Distance Between Motif IV, Ia, and V and the Nearest ssRNA Phosphates (P) for Clusters ^R^M1, ^R^M2, ^R^M3, and ^R^M4 in L405D Mutated nsp13 and ^R^W1, ^R^W2, ^R^W3, Wild-Type nsp13, Respectively^a^

	average distance (Å)						
	L405D MT nsp13				WT nsp13		
residue	^R^M1	^R^M2	^R^M3	^R^M4	^R^W1	^R^W2	^R^W3
IV–P	5.5(1)	5.3(1)	6.6(9)	5.8(1)	0	5.2(2)	3.8(2)
Ia-P	7.0(1)	6.0(6)	6.5(1)	5.7(8)	0	6.6(3)	4.7(5)
V–P	6.6(6)	5.6(5)	5.9(5)	6.4(6)	0	6.3(0)	4.4(3)

a Error in the last digit is provided in parentheses.

**Table 2:** Motif-Specific Closest Phosphates: Reweighted Probability for Each RNA-Cleft Cluster in L405D Mutated nsp13

	probability			
residue	^R^M1(%)	^R^M2(%)	^R^M3(%)	^R^M4(%)
IV–P2	77	97	85	94
IV–P3	29	30	19	18
Ia-P4	37	96	58	33
Ia-P5	63	9	46	67
V–P3	96	94	83	94
V–P4	5	21	39	8

Collectively, these RNA-cleft states demonstrate that L405D introduces extraneous binding configurations that scramble the strict alternation of tight/loose contacts required for directional stepping. Instead of a single, ATP-synchronized translocation event per cycle, the mutant samples an ^R^M1 →^R^M3 →^R^M1 →^R^M2 sequence that, based on the associated motif–RNA registers, does not correspond to the productive forward progression proposed for wild type. This altered conformational progression provides a potential structural explanation for the experimentally observed attenuation of helicase activity^27^ and supports an important role for the RNA cleft as an allosteric hub coupling nucleotide chemistry to mechanical translocation. These findings suggest that stabilizing non-productive cleft conformations may represent a promising antiviral strategy.

### L405D Reshapes the ATP-Dependent Conformational Ensemble

Unlike wild-type nsp13, which relies on both conformational selection and ATP-induced conformational induction,^16,19,49^ the ATP cycle of the L405D mutant demonstrates only conformational selection. In the wild-type helicase, ATP binding and product release drive a directional progression through four dominant protein conformational clusters (^P^W1–^P^W4; Figure 4b,d), where some states are shared across hydrolysis intermediates through conformational selection and others are newly accessed only upon ATP binding or product release through conformational induction.^16^ In particular, ATP binding induces sampling of the ^P^W3 cluster, which is absent in the nucleotide-free ensemble, while the ADP-bound state preferentially accesses a distinct ^P^W4 cluster that is not populated in the ATP-bound state. These induced conformations have been proposed to contribute to coupling nucleotide turnover to the domain rearrangements associated with ATP-pocket opening, product release, and RNA translocation. The behavior of the L405D mutant differs fundamentally from this mechanism, indicating that the mutation does not simply shift populations but instead alters the underlying mode of conformational regulation.

**Figure 4:**
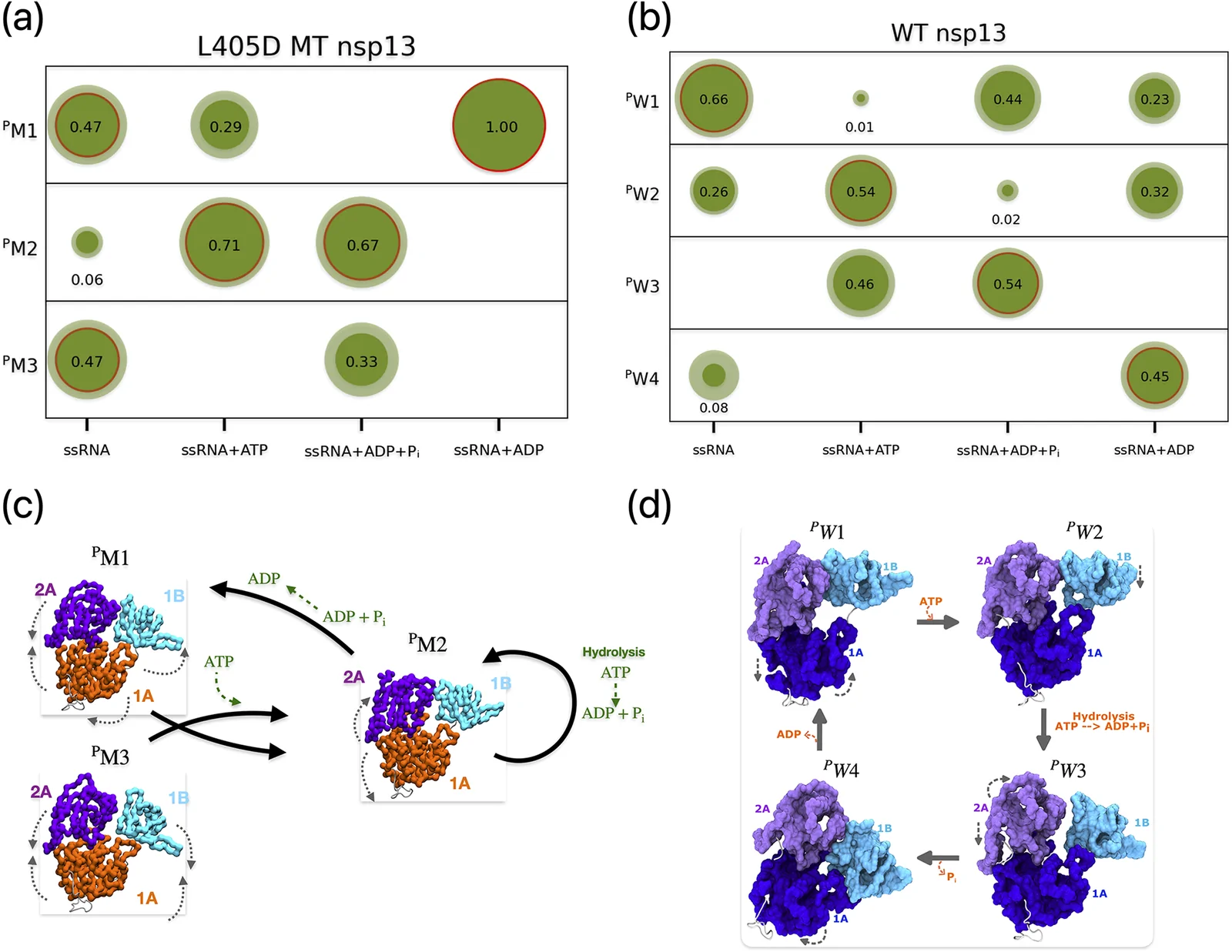
Full protein nsp13 conformational clusters using Shape-GMM. (a) and (b) The reweighted probability of clusters explored by L405D mutated (MT) nsp13 and wild type (WT) nsp13 system are plotted here and presented in scatter form. The size of each circle is proportional to the respective cluster’s probability and the surrounding halo represents the associated uncertainty estimated by the 2nd-order cumulant reweighting. (c, d) Conformational clusters of MT and WT nsp13 across ssRNA, ssRNA+ATP, ssRNA+ADP+P_*i*_, ssRNA+ADP ensembles, as determined by Shape-GMM clustering on the C_*α*_ positions of domains 1B, 1A, and 2A.

L405D reduces the conformational diversity accessible during the ATP hydrolysis cycle, collapsing the four-state wild-type landscape into three dominant states. To characterize mutation-dependent changes in the global conformational ensemble, clustering was performed using the C_*α*_ positions of the 1A, 1B, and 2A domains across the combined ssRNA-, ATP-, ADP+P_*i*_-, and ADP-bound ensembles. The optimal number of mutant protein conformational clusters was determined to be three (^P^M1–^P^M3) based on the plateau in log-likelihood improvement and the minimum in the second derivative of the training-set curve (Figure S3), in contrast to the four-cluster landscape previously identified for wild-type nsp13. Linear discriminant analysis further shows that these mutant clusters are well separated but reorganized relative to wild type (Figure 4a): LD1 primarily separates ^P^M2 from the remaining ensemble, while LD2 distinguishes ^P^M1 from ^P^M3. This partitioning differs qualitatively from the wild-type system, where ATP-dependent sampling of four distinct clusters reflects a broader conformational repertoire. As shown in Figure 4c, the cluster representatives reveal that L405D samples a reduced conformational space, suggesting restricted access to states required for efficient progression through the ATP hydrolysis cycle.

No hydrolysis state in the L405D mutant accesses a uniquely induced conformation absent from the apo ensemble, indicating loss of ATP-driven conformational induction. In the ssRNA-only state, the conformational ensemble is split equally between ^P^M1 and ^P^M3 (0.47 ± 0.29 each), establishing these states as the principal nucleotide-free conformations (Figure 4b). ATP binding preserves ^P^M1 (0.29 ± 0.26) while shifting the dominant population toward ^P^M2 (0.71 ± 0.26), but critically does not introduce any cluster absent from apo ensemble, the hallmark signature of conformational induction is entirely absent. The ADP+P_*i*_–bound state retains ^P^M2 as the dominant state (0.67 ± 0.33) with residual ^P^M3 (0.33 ± 0.33), yet without accessing a new induced conformation. Following phosphate release, the ADP-bound state collapses entirely into ^P^M1 (1.0 ± 0.0), returning to the same dominant nucleotide-free conformation rather than accessing a distinct post-hydrolysis state as seen in wild-type.^16^ The collapse of the ADP-bound state into ^P^M1 indicates that product release provides no conformational driving force for resetting the enzyme toward a new catalytic cycle. In contrast to wild-type nsp13, where ATP binding and product release induce sampling of conformations not present in the nucleotide-free state, every hydrolysis state of the L405D mutant remains confined to the same pre-existing set of clusters. These observations demonstrate that substrate binding drives only population redistribution among existing states, the hallmark of conformational selection, while the ATP-induced conformational induction required for productive catalytic cycling is lost.

The loss of conformational induction suggests that L405 acts as a mechanistic pivot that couples ATP binding to productive domain rearrangement in nsp13. In the wild-type helicase, linear discriminant analysis of the protein conformational clusters showed that L405 contributes most strongly to the dominant ATP-dependent opening and closing motion between the 1A, 1B, and 2A domains, highlighting its central role in organizing the conformational response to substrate binding.^16^ Substitution of this residue with aspartate does not simply shift equilibrium populations within the existing ensemble; within the sampled ensemble, ATP binding and product release do not induce access to clusters absent from the nucleotide-free state. The collapse of the ADP-bound state entirely into ^P^M1 is particularly telling: rather than the enzyme advancing to a new post-hydrolysis conformation that facilitates product release and reset, it reverts to the apo-like PM1 state while ADP remains bound, a conformational pattern that we hypothesize may be unfavorable for efficient product release and catalytic reset. This result is consistent with reduced access to structural transitions associated with ATP-pocket opening, coordinated RNA-cleft rearrangement, and efficient product release. The L405D mutation converts a directional four-state catalytic cycle into a restricted three-state ensemble governed by conformational selection.

To determine whether L405D stabilizes genuinely novel conformations or merely redistributes population among wild-type-accessible states, we projected both trajectories onto the mutant- and wild-type-derived LD axes and compared their reweighted probability distributions (Figure S6). The Jensen–Shannon divergence between the two ensembles is large along both mutant-derived axes (*JS*_LD1_ = 0.69, *JS*_LD2_ = 0.69) and the wild-type-derived axis (*JS* = 0.62), with near-zero distributional overlap coefficients (*OC* < 0.03) across all projections. JS divergence values above 0.5 indicate ensembles that are more different than alike, and the consistency of this result across both coordinate systems rules out a coordinate artifact. These findings demonstrate that L405D does not redistribute population among pre-existing wild-type states but instead stabilizes a conformational landscape that is largely inaccessible to the wild-type enzyme, demonstrating that the altered nucleotide-dependent behavior of L405D occurs within a conformational landscape distinct from that sampled by wild type.

The loss of conformational induction suggests that ATP-pocket opening and product release should be kinetically impaired in the L405D mutant. In wild-type nsp13, ATP binding and hydrolysis drive access to induced conformations that facilitate coordinated opening and closing of the 1A–2A ATP-binding cleft, enabling efficient ATP binding, phosphate release, and eventual ADP dissociation. When this inductive component^21^ is lost, the enzyme becomes restricted to redistribution among pre-existing states, increasing the population of closed or partially closed conformations that may be less favorable for product release. The complete collapse of the ADP-bound state into ^P^M1 further suggests that the conformational memory required to distinguish apo from product-bound states is erased, suggesting reduced ability to couple ADP dissociation to the domain rearrangements needed to prime the next catalytic cycle. This provides a structural explanation for the experimentally observed attenuation of ATPase turnover^27^ despite preserved ATP binding and catalytic competence.^50^ We therefore next examine the specific interactions that underlie the observed behavior.

### Protein Clustering Reveals Specific Residue Interactions

No significant large-scale interdomain differences were observed between the wild-type and L405D ensembles. This is consistent with the experimental observation that L405D retains measurable ATPase and helicase activity despite attenuation of both functions.^27^ See the discussion of interdomain angles in the Supporting Information for more details. There are, however, structural differences observed at the residue level.

To identify the specific residues demonstrating conformational differences in the L405D ensemble, we decomposed the linear discriminant (LD) vectors from LDA into per-residue contributions and mapped their normalized magnitudes onto the protein structure (Figure S6). Along LD1, the highest-contributing residues are concentrated at the 1B–1A domain interface, with dominant peaks at residues 177–178 (domain 1B) and 308–309 (domain 1A), alongside a cluster of high-magnitude residues at 374–376 (domain 1A) and residue 514 (domain 2A), consistent with coordinated 1A–1B and 1A–2A cleft rearrangements identified above. Along LD2, discriminating residues localize predominantly to the 1A and 2A domains, with the highest contributions at residue 177 (domain 1B), residues 283 and 375 at the 1A–2A interface, residues 443–444 at the 1A–2A domain boundary, and residues 485, 515, 556–567 within domain 2A, reflecting the independent RNA-cleft rearrangements observed across the hydrolysis cycle. Notably, the elevated LD magnitudes observed near the 1A–2A domain boundary (residues 443–444) and within domain 2A (residues 514–515) along both discriminants suggest that the conformational discrimination between clusters originates from a localized structural perturbation in the vicinity of Motif V that propagates outward to the inter-domain interfaces.

We next examined whether these long-range conformational changes are associated with perturbation of the Motif V salt-bridge network introduced by the L405D substitution. In our previous study and recent experimental work on Motif V,^16,27^ L405 was proposed to function as an allosteric regulator by positioning the D534–R560 salt bridge that couples the ATPase site to the RNA-binding cleft. Replacing L405 with aspartate introduces a new negatively charged residue adjacent to D534, potentially altering the local electrostatic environment surrounding R560.

Our distance analysis is consistent with perturbation of this interaction network (Table 3). In both the ^P^M1 and ^P^M2 clusters, D534 maintains close contact with R560 (4.4–5.5 Å), while D405 remains more distant (6.2–6.9 Å), consistent with preferential stabilization of the native D534–R560 interaction throughout the accessible conformational ensemble. Importantly, neither cluster supports a configuration in which D405 competitively displaces D534 at R560; instead, the mutant residue remains peripherally positioned in both dominant states. Thus, the effect of the L405D substitution appears to involve perturbation of the local electrostatic environment rather than direct replacement of the native D534–R560 interaction. We propose that this perturbation may contribute to the long-range changes in domain coordination, ATP-pocket accessibility, and RNA-cleft synchronization observed in the mutant (Table 4).

**Table 3:** Reweighted Average of OD1–NH1 Salt-Bridge Distances (Å) between D534–R560 and D405–R560 Across Predominant Structural Clusters of L405D Mutated nsp13 (^P^M1–^P^M3)^a^

	D534-R560 (OD1-NH1)	D405-R560 (OD1-NH1)
^P^M1	4.4(6)	6.9(1)
^P^M2	5.5(8)	6.2(9)
^P^M3	4.6(5)	4.9(6)

a Values are reported as mean (standard deviation).

**Table 4:** Reweighted Average of OD1–NH1 Salt-Bridge Distances (Å) between D534–R560 Across Predominant Structural Clusters of L405D Mutated nsp13 (^P^M1 – ^P^M3) and Wild Type (WT) nsp13 (^P^W1–^P^W4)^a^

	L405D MT nsp13			WT nsp13			
cluster	^P^M1	^P^M2	^P^M3	^P^W1	^P^W2	^P^W3	^P^W4
D534-R560 (OD1-NH1)	4.4(6)	5.5(8)	4.6(5)	3.1(4)	3.5(3)	3.2(2)	3.3(4)

a Values are reported as mean (standard deviation).

## Conclusions

This work provides a molecular mechanism for how the L405D mutation decouples ATP hydrolysis from productive RNA translocation in the SARS-CoV-2 nsp13 helicase. Experimentally, L405D attenuates both ATPase and helicase activities, with a substantially stronger effect on helicase function. Our simulations provide a molecular model for this phenotype in which altered conformational sampling disrupts coordination between nucleotide state and RNA-cleft rearrangement. At the protein-ensemble level, we do not observe nucleotide-induced access to conformational clusters absent from the nucleotide-free ensemble, in contrast to the four-state wild-type landscape. Based on the cluster characterizations and populations along the ATP hydrolysis cycle, a coherent ATP-dependent translocation mechanism could not be identified. This nucleotide-insensitive behavior is consistent with a reduction in ATP-dependent helicase efficiency observed in the mutant as compared to the wild-type.

At the RNA-cleft level, the mutant samples a proposed ^R^M1→^R^M3→^R^M1→^R^M2 sequence in which the forward register shift initiated upon ATP binding is reversed by hydrolysis rather than consolidated, and the ADP-bound ensemble strongly populates a tight-grip conformation that may be unfavorable for RNA release and productive reset. The canonical motif IV–Ia two-phosphate gap required for directional stepping is never preserved across the cycle. At the molecular level, these effects are associated with perturbation of the native D534—R560 electrostatic network within Motif V, which is associated with long-range changes in ATP-pocket accessibility and RNA-cleft synchronization without abolishing catalytic activity.

Together, these findings support L405 as a likely mechanistic pivot coupling ATP-driven conformational induction to directional translocation in nsp13, and support an important role for the RNA-binding cleft as an allosteric hub where chemical and mechanical cycles are coordinated. The non-productive RNA-cleft conformations identified here may provide a conceptual basis for future efforts to develop allosteric inhibitors that stabilize such states.

## Supplementary Material



## Data Availability

The data that support the findings of this study, including simulation input files, analysis scripts, and cluster ID files, are contained in the GitHub repo https://github.com/mccullaghlab/L405D_Analyses. Raw trajectory files are available from the corresponding author upon reasonable request.

## Notes

Additional details of the molecular dynamics simulations and conformational analyses, including simulation and clustering statistics, replica-specific cluster populations, Shape-GMM clustering validation, motif–RNA contacts and distances, interdomain cleft-angle analyses, and statistical characterization of the conformational ensembles (PDF)
