# Supplementary material for: Mechanochemical Decoupling of ATP Hydrolysis and RNA Translocation in SARS-CoV‑2 nsp13 by the L405D Mutation
Source: J Phys Chem B. 2026 Sep 9;130(38):9851–61. doi: 10.1021/acs.jpcb.6c03935 (PMC13618043; doi:10.1021/acs.jpcb.6c03935)
Supplement: Supplementary file 1 [file jp6c03935_si_001.pdf]

Supporting Information for: Mechanochemical Decoupling of ATP Hydrolysis and RNA Translocation in SARS-CoV-2 nsp13 by the L405D Mutation

Elham Fazelpour, Priti Roy, Kole J. Frederick, and Martin McCullagh*

Department of Chemistry, Oklahoma State University, Stillwater, OK 74078, USA

E-mail: martin.mccullagh@okstate.edu

S1 Supporting Table and Figures

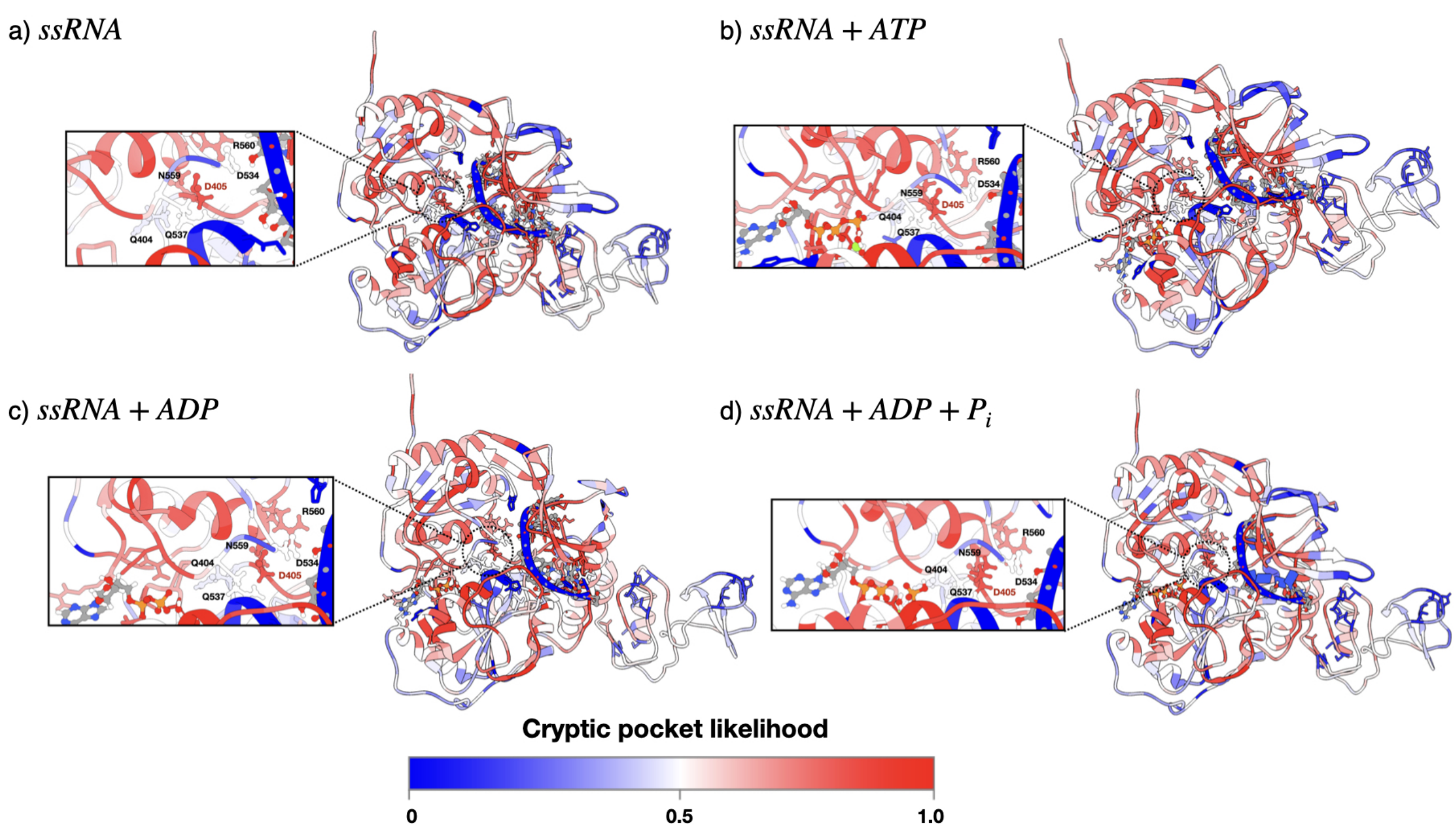

Figure S1: PocketMiner predictions on each substrate state. Proteins are colored from blue to red as predictions range from negative

Table S1: Summary of MD simulations and ShapeGMM-based cluster frame selection for the ssRNA, ssRNA+ATP, ssRNA+ADP+P_i, and ssRNA+ADP systems.

system	replicas	cMD[a] length (ns)	GaMD[b] length (μs)	GaMD frames per replica	protein clustering[c] frames per replica	RNA clustering frames per replica
ssRNA	3	10	3	300k	30k	300k
ssRNA+ATP	3	10	3	300k	30k	300k
ssRNA+ADP+P_i	3	500	3	300k	30k	300k
ssRNA+ ADP	3	10	3	300k	30k	300k

[a] conventional molecular dynamics
[b] Gaussian accelerated molecular dynamics
[c] Protein clustering was performed using every 10th frame from the GaMD production trajectory (stride = 10).

Table S2: Replica breakdown of RNA cluster populations.

Cluster ID	ssRNA			ssRNA+ATP			ssRNA+ADP+P_i			ssRNA+ADP		
	rep 1	rep 2	rep 3	rep 1	rep 2	rep 3	rep 1	rep 2	rep 3	rep 1	rep 2	rep 3
^{R}M1	0.37	0.88	0.91	0.10	0.03	0.18	0.49	0.73	0.40	0.00	0.01	0.59
^{R}M2	0.23	0.00	0.01	0.37	0.04	0.07	0.01	0.14	0.17	0.95	0.98	0.10
^{R}M3	0.03	0.02	0.05	0.12	0.72	0.56	0.32	0.02	0.36	0.00	0.00	0.03
^{R}M4	0.37	0.10	0.04	0.42	0.21	0.20	0.19	0.12	0.06	0.05	0.01	0.29

Table S3: Replica breakdown of Protein cluster populations.

Cluster ID	ssRNA			ssRNA+ATP			ssRNA+ADP+P_i			ssRNA+ADP		
	rep 1	rep 2	rep 3	rep 1	rep 2	rep 3	rep 1	rep 2	rep 3	rep 1	rep 2	rep 3
^{P}M1	0.00	0.99	0.42	0.81	0.00	0.07	0.00	0.00	0.00	1.00	1.00	1.00
^{P}M2	0.00	0.01	0.18	0.19	1.00	0.93	1.00	1.00	0.00	0.00	0.00	0.00
^{P}M3	1.00	0.00	0.41	0.00	0.00	0.00	0.00	0.00	1.00	0.00	0.00	0.00

Table S4: Replica breakdown of RNA cluster populations in Wild type system.

Cluster ID	ssRNA			ssRNA+ATP				ssRNA+ADP+P_i			ssRNA+ADP			
	rep 1	rep 2	rep 3	rep 1	rep 2	rep 3	rep 4	rep 1	rep 2	rep 3	rep 1	rep 2	rep 3	rep 4
^{R}W1	0.47	0.89	0.0	0.08	0.83	0.47	0.53	0.00	0.00	0.03	0.96	0.51	0.70	0.60
^{R}W2	0.48	0.1	0.21	0.44	0.13	0.34	0.47	0.19	0.09	0.14	0.03	0.42	0.26	0.39
^{R}W3	0.05	0.02	0.79	0.45	0.04	0.19	0.00	0.81	0.91	0.83	0.01	0.07	0.03	0.01

Table S5: Average RNA Cluster Populations with Standard Error from Replicas in Wild type system.

Cluster ID	ssRNA	ssRNA+ATP	ssRNA+ADP+P_i	ssRNA+ADP
^{R}W1	0.45 ± 0.26	0.48 ± 0.15	0.01 ± 0.01	0.70 ± 0.10
^{R}W2	0.26 ± 0.11	0.35 ± 0.08	0.14 ± 0.03	0.28 ± 0.09
^{R}W3	0.28 ± 0.25	0.18 ± 0.11	0.85 ± 0.03	0.03 ± 0.01

Table S6: Protein components populations for each replica in Wild type system

Cluster ID	ssRNA			ssRNA+ATP				ssRNA+ADP+P_i			ssRNA+ADP			
	rep 1	rep 2	rep 3	rep 1	rep 2	rep 3	rep 4	rep 1	rep 2	rep 3	rep 1	rep 2	rep 3	rep 4
^{P}W1	0.00	0.17	0.07	0.17	0.75	0.77	0.74	0.00	0.00	0.00	0.45	0.61	0.84	0.59
^{P}W2	0.50	0.52	0.80	0.80	0.00	0.00	0.00	0.00	0.00	0.00	0.09	0.07	0.01	0.01
^{P}W3	0.50	0.30	0.11	0.04	0.25	0.23	0.26	0.00	0.00	0.00	0.46	0.32	0.15	0.40
^{P}W4	0.00	0.00	0.03	0.00	0.00	0.00	0.00	1.00	1.00	1.00	0.00	0.00	0.00	0.00

Table S7: Average Protein Cluster Populations with Standard Error from Replicas in Wild type system

Cluster ID	ssRNA	ssRNA+ATP	ssRNA+ADP+P_i	ssRNA+ADP
^{P}W1	0.08 ± 0.05	0.61 ± 0.15	0.00 ± 0.00	0.62 ± 0.08
^{P}W2	0.61 ± 0.09	0.20 ± 0.20	0.00 ± 0.00	0.04 ± 0.02
^{P}W3	0.30 ± 0.11	0.20 ± 0.05	0.00 ± 0.00	0.33 ± 0.07
^{P}W4	0.01 ± 0.01	0.00 ± 0.00	1.00 ± 0.00	0.00 ± 0.00

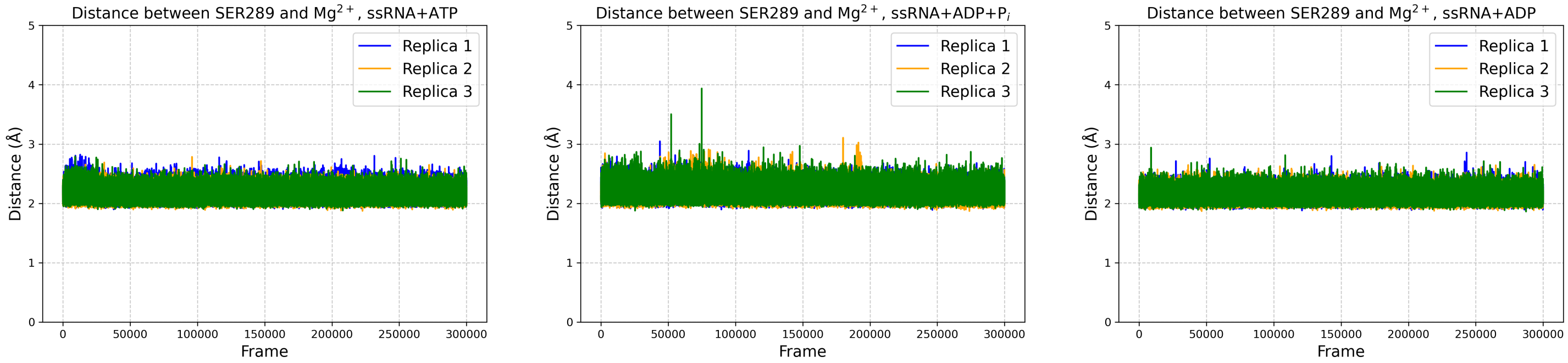

(a) ssRNA+ATP (b) ssRNA+ADP+P_i (c) ssRNA+ADP

Figure S2: Mg^{2+} coordination stability with SER289 across nucleotide states during GaMD production simulations. Distance between the catalytic Mg^{2+} ion and SER289 as a function of simulation frame number for the nsp13–ssRNA complex in the (A) ATP-, (B) ADP+P_i-, and (C) ADP-bound states. Each panel shows three independent GaMD production replicates (n = 3), distinguished by color as indicated in the legend. The Mg^{2+}– SER289 distance remains stable across all frames and replicates, averaging 2.0–2.5 Å, consistent with sustained octahedral Mg^{2+} coordination geometry and supporting the structural integrity of the modeled active sites.

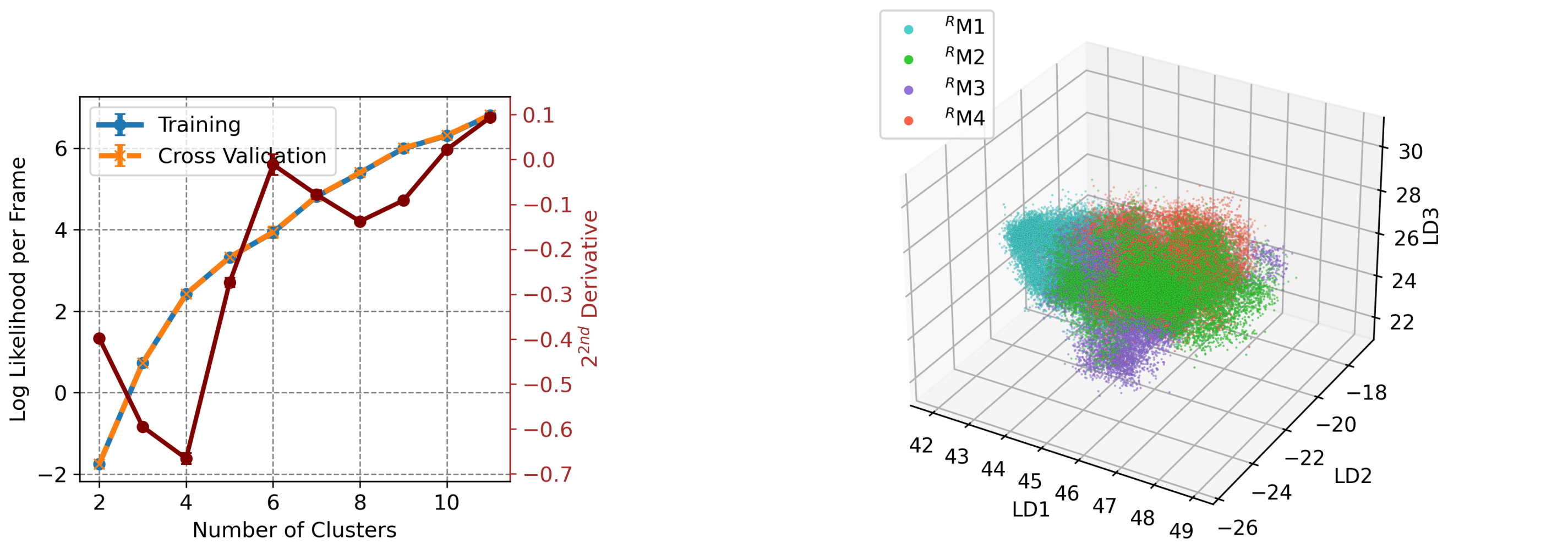

(a) (b)

Figure S3: RNA cleft conformational clusters were determined by shape-GMM clustering. (a) Log likelihood per frame as a function of the number of clusters is plotted for the training (blue) and CV (orange) sets using the weighted Shape-GMM method. The change on slope of training set computed by the second derivative is plotted in red with values given on the right-hand y-axis. We used 80% of 3.6 million frames for training. (b) Projection of the amalgamated trajectory onto the three linear discriminants (LD1, LD2, and LD3) determined using LDA on Shape-GMM clusters.

Table S8: Motifs Ia, IV, and V residues in contacts with RNA “phosphates (P)” around 5.0 Å for SF1 RNA-bound helicases protein crystal structures and the percentage (%) of frames where the corresponding nsp13 residues are in contact with RNA(P) for each simulated substrate state.

Motif	upf1 (2XZL)	IGHMBP2 (4B3G)	Nsp13 (7NN0)	ssRNA	ssRNA+ATP	ssRNA+ADP+P_i	ssRNA+ADP
Ia	PRO460	PRO243	CYS309	2.5	55.8	36.4	17.4
Ia	SER461	SER244	SER310	31.6	88.1	55.1	52.7
Ia	ASN462	ASN245	HIS311	71.1	91.7	72.9	95.9
Ia	VAL463	ILE246	ALA312	14.9	9.7	6.2	12.0
IV	PRO731	PRO540	PRO514	41.8	24.5	19.4	16.5
IV	TYR732	TYR541	TYR515	100	69.0	70.7	100
IV	GLU733	ASN542	ASN516	100	47.8	63.6	100
IV	GLY734	LEU543	SER517	68.2	30.3	35.6	86.1
V	ALA764	ASP565	ASP534	59.0	96.4	97.9	73.5
V	SER761	SER563	SER535	65.3	33.3	61.3	93.1

Table S9: Residues of motifs in which C_α positions are used for distance calculations.

Motif	Residue
Ia	HIS311
IV	ASN516
V	ASP534

Table S10: Replica breakdown of the average reweighted separation distances in L405D mutated nsp13 between Motifs IV, Ia, and V and the nearest ssRNA phosphates (P) for clusters ^{R}M1, ^{R}M2, ^{R}M3, and ^{R}M4. Errors in the last digit are given in parentheses.

substrate	cleft	^{R}M1			^{R}M2			^{R}M3			^{R}M4		
		rep1	rep2	rep3	rep1	rep2	rep3	rep1	rep2	rep3	rep1	rep2	rep3
ssRNA	IV-P	4.6(1)	5.0(2)	4.6(2)	4.8(1)	-	4.6(0)	4.5(0)	4.8(0)	-	4.9(2)	4.5(0)	4.8(0)
	Ia-P	5.1(2)	8.5(5)	6.7(7)	6.5(9)	-	5.2(3)	5.1(1)	6.9(1)	-	4.9(0)	7.4(4)	6.1(6)
	V-P	5.7(6)	7.3(5)	6.0(6)	5.9(0)	-	6.0(3)	5.6(0)	8.0(3)	-	5.5(0)	7.7(2)	6.1(0)
ssRNA + ATP	IV-P	4.6(2)	7.2(2)	7.3(4)	4.8(1)	7.3(7)	9.1(3)	-	7.4(1)	7.0(2)	4.7(0)	7.1(3)	8.5(6)
	Ia-P	5.4(0)	5.0(1)	8.3(6)	5.5(1)	5.3(2)	6.6(1)	-	5.4(0)	7.4(8)	5.4(2)	5.0(0)	5.6(8)
	V-P	5.7(2)	5.8(3)	7.4(5)	6.1(5)	5.8(2)	5.2(0)	-	5.7(1)	5.7(0)	6.3(1)	5.7(2)	6.7(1)
ssRNA + ADP +P_i	IV-P	5.4(9)	8.0(1)	4.8(0)	7.0(2)	9.5(6)	-	6.6(7)	7.4(4)	4.8(2)	6.5(1)	9.6(9)	4.9(1)
	Ia-P	6.7(7)	6.1(5)	8.4(5)	7.4(6)	6.0(3)	-	5.5(6)	6.1(4)	8.1(6)	5.4(4)	6.7(5)	8.9(4)
	V-P	6.6(1)	6.8(5)	6.0(0)	8.2(3)	6.7(3)	-	6.8(7)	6.8(3)	5.7(2)	6.7(4)	6.9(4)	5.6(2)
ssRNA + ADP	IV-P	-	-	5.1(2)	5.2(0)	4.8(1)	4.8(2)	5.2(0)	-	-	4.8(2)	-	4.9(2)
	Ia-P	-	-	6.6(3)	6.6(9)	5.5(3)	6.4(4)	5.8(0)	-	-	6.6(2)	-	6.6(0)
	V-P	-	-	7.3(3)	5.5(3)	5.1(3)	7.0(5)	5.0(2)	-	-	5.6(2)	-	7.2(1)

Table S11: Replica breakdown of motif-specific closest Phosphates reweighted probability(%) in each substrate of L405D mutated nsp13.

substrate	residue	^{R}M1			^{R}M2			^{R}M3			^{R}M4		
		rep1	rep2	rep3	rep1	rep2	rep3	rep1	rep2	rep3	rep1	rep2	rep3
ssRNA	IV-P2	100	60	84	100	0	100	100	61	0	100	95	100
	IV-P3	0	40	16	0	0	0	0	39	0	0	5	0
	Ia-P4	87	20	57	88	0	24	15	99	0	5	65	29
	Ia-P5	13	80	43	12	0	76	85	1	0	95	4	71
	V-P3	99	100	4	96	0	100	12	100	0	100	100	100
	V-P4	1	0	2	4	0	0	88	0	0	0	0	0
ssRNA + ATP	IV-P2	100	100	66	100	100	54	0	100	100	100	100	97
	IV-P3	0	0	34	0	0	46	0	0	0	0	0	3
	Ia-P4	64	100	0	100	100	13	0	100	27	61	96	4
	Ia-P5	36	0	95	0	0	87	0	0	73	39	4	96
	V-P3	100	100	75	100	100	55	0	100	69	100	100	60
	V-P4	0	0	25	0	0	45	0	0	31	0	0	40
ssRNA + ADP +P_i	IV-P2	32	76	100	0	53	0	3	70	100	52	88	100
	IV-P3	68	24	0	100	47	0	97	30	0	48	12	0
	Ia-P4	20	0	37	0	0	0	14	0	64	23	0	66
	Ia-P5	80	100	63	100	100	0	86	100	36	77	100	34
	V-P3	84	94	100	15	11	0	49	100	100	84	89	100
	V-P4	16	6	0	85	89	0	51	0	0	16	11	0
ssRNA + ADP	IV-P2	0	0	100	100	100	100	100	0	0	100	0	100
	IV-P3	0	0	0	0	0	0	0	0	0	0	0	0
	Ia-P4	0	0	64	100	99	99	100	0	0	0	0	1
	Ia-P5	0	0	36	0	1	1	0	0	0	100	0	99
	V-P3	0	0	100	100	100	100	100	0	0	100	0	100
	V-P4	0	0	0	0		0	0	0	0	0	0	0

Table S12: Replica breakdown of reweighted values of angles between subdomains for each protein cluster in L405D mutated nsp13. Error in the last digit is provide in parentheses

substrate	cleft	^{P}M1			^{P}M2			^{P}M3		
		rep1	rep2	rep3	rep1	rep2	rep3	rep1	rep2	rep3
ssRNA	1A-2A(θ_1)	-	46(0)	47(1)	-	-	49(1)	46(1)	-	48(1)
	1A-1B(θ_2)	-	76(1)	72(2)	-	-	69(1)	67(1)	-	70(1)
	2A-1B(θ_3)	-	56(1)	62(2)	-	-	63 (1)	66(1)	-	62(0)
ssRNA + ATP	1A-2A(θ_1)	45(1)	-	-	47(0)	46(1)	46(2)	-	-	-
	1A-1B(θ_2)	72(1)	-	-	69(0)	71(1)	72(2)	-	-	-
	2A-1B(θ_3)	63(1)	-	-	63(0)	63(1)	63(1)	-	-	-
ssRNA +ADP +P_i	1A-2A(θ_1)	-	-	-	46(1)	51(0)	-	-	-	44(1)
	1A-1B(θ_2)	-	-	-	71(2)	69(1)	-	-	-	67(2)
	2A-1B(θ_3)	-	-	-	63(3)	60(1)	-	-	-	67(3)
ssRNA +ADP	1A-2A(θ_1)	43(1)	44(0)	46(1)	-	-	-	-	-	-
	1A-1B(θ_2)	77(2)	72(1)	73(1)	-	-	-	-	-	-
	2A-1B(θ_3)	59(1)	64(1)	61(1)	-	-	-	-	-	-

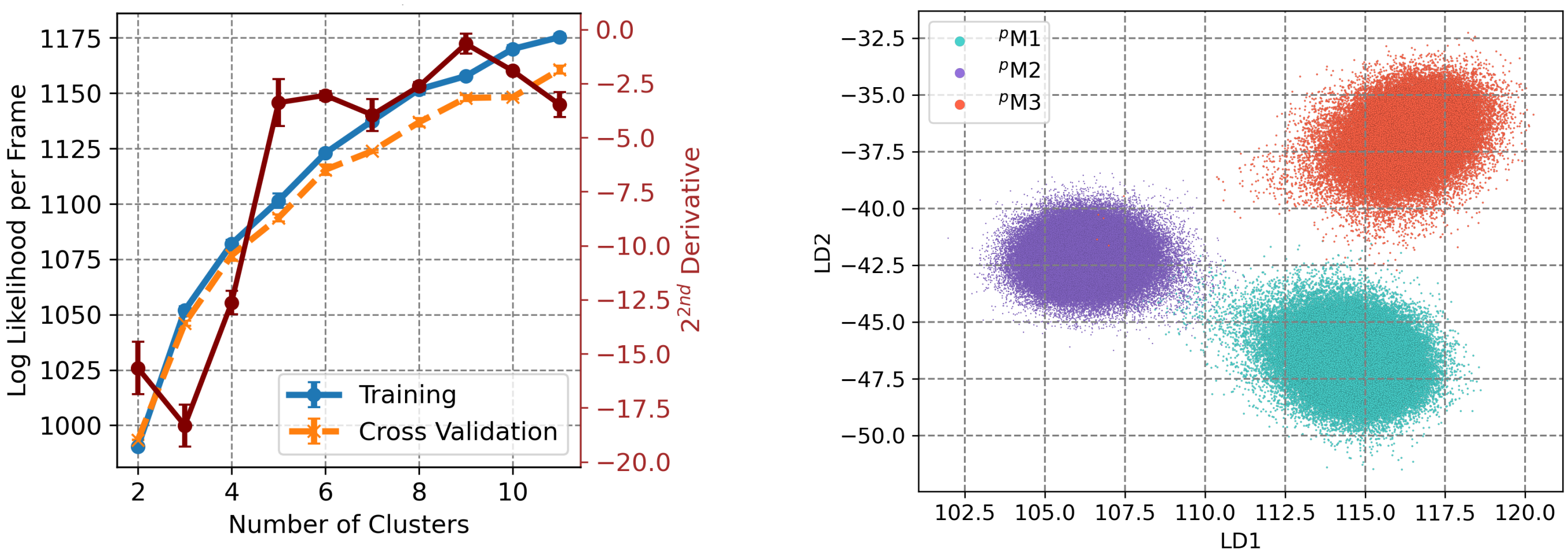

(a) (b)

Figure S4: Identification of unique protein conformational clusters within the ensemble of nsp13-bound ssRNA, ATP+ATP, ssRNA+ADP+P_i, ssRNA+ADP, determined by Shape-GMM at the C_α resolution of domains 1B, 1A, and 2A. (a) Log likelihood per frame as a function of the number of clusters is plotted for the training (blue) and CV (orange) sets using the weighted Shape-GMM method. The change on slope of training set computed by the second derivative is plotted in red with values given on the right-hand y-axis. We used 80% of ∼300k frames for training. (b) The 2D scatter plot denotes the projection of the amalgamated trajectory onto LD1 and LD2 eigenvectors determined using LDA on Shape-GMM clusters.

S2 Analyses

Inter-domain Angle Discussion

To quantify interdomain geometries, we analyzed the the reweighted probability distributions and mean values of the angles formed by vectors connecting the centers of mass of the three major domains (Figure S5a). The angle θ_1 reports on the 1A–2A interdomain cleft that defines the ATP-binding pocket, while θ_2 and θ_3 describe the two RNA-cleft interfaces between 1A–1B and 2A–1B, respectively. Pairwise Welch's t-tests confirmed that the three conformational clusters are statistically distinct across all three angle dimensions ($p < 0.001$ for most comparisons; see next subsection and Figure S6 and S7), supporting the conclusion that these clusters represent genuinely distinct conformational states rather than arbitrary partitions of a continuous distribution (Figure S6).

Inter-domain Cleft Angle Statistics

To characterize the conformational differences between the three protein clusters (^{P}M1, ^{P}M2, ^{P}M3) identified by shapeGMM, the inter-domain cleft angles θ_1 (1A-2A), θ_2 (1A-1B), and θ_3 (2A-1B) were computed for each frame of the trajectory. Each angle was defined as the angle subtended at one domain's center of mass by vectors pointing toward the centers of mass of the other two domains, computed using the dot product of the normalized inter-domain vectors:

$$\theta = \arccos\left(\frac{\mathbf{v}_1 \cdot \mathbf{v}_2}{|\mathbf{v}_1||\mathbf{v}_2|}\right) \tag{1}$$

The centers of mass were calculated for three protein domains: domain 1B (residues 151–226), domain 1A (residues 260–441), and domain 2A (residues 444–601), using only C_α atoms.

To account for the enhanced sampling bias introduced during the simulation, average angles were computed for each cluster using the second-order cumulant expansion (CE2) reweighting method, applied here with adaptive equal-population angle bins (≥ 100 frames/bin) rather than direct per-frame exponential reweighting, since the latter is numerically unstable given the heavy-tailed GaMD boost distribution.[1] Reweighted means and 95% confidence intervals (non-overlapping block bootstrap, 1000-frame blocks, 200 resamples[2]) were computed identically for the bar-plot and probability-distribution analyses below.

Because MD frames within a replica are highly autocorrelated, raw frame counts substantially overstate

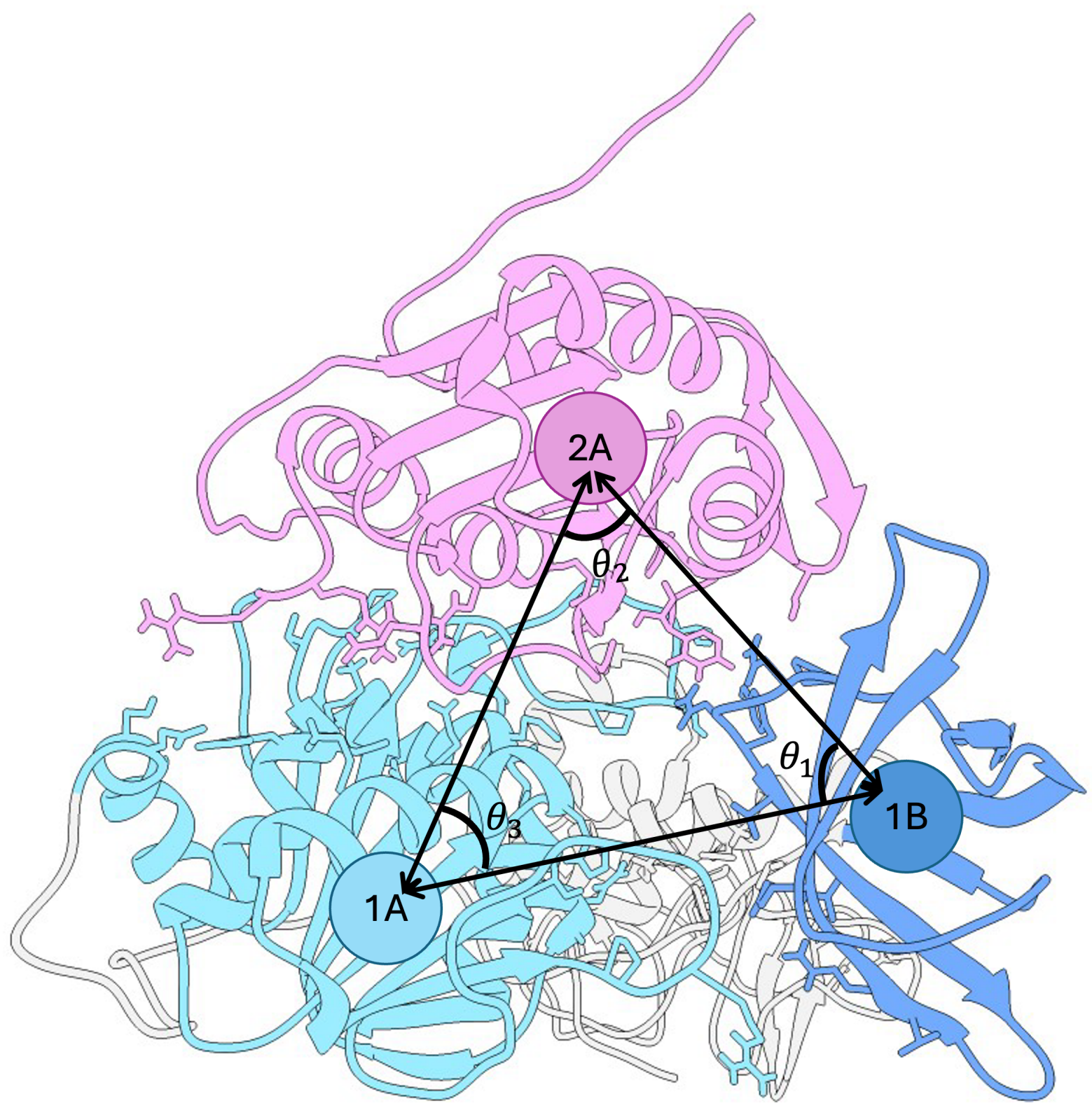

(a)

	Average Angle(deg)						
	L405D MT nsp13			WT nsp13			
cleft	^{P}M1	^{P}M2	^{P}M3	^{P}W1	^{P}W2	^{P}W3	^{P}W4
1A-2A(θ_1)	45(1)	47(3)	45(4)	44(1)	45(1)	46(1)	46(2)
1A-1B(θ_2)	73(2)	70(2)	67(2)	70(0)	72(4)	72(3)	70(2)
2A-1B(θ_3)	61(5)	63(3)	66(1)	66(1)	63(3)	63(2)	64(6)

(b)

Figure S5: Interdomain angles within the nsp13 structure and the calculated reweighted average values in each protein cluster. (a) The spheres denote the centers of mass of the respective domains, and the black arrows represent the separation vectors between them. The arrow directions are defined relative to each specific angle calculation; for example, in computing θ_1, the vectors are oriented from domain 1B toward domains 1A and 2A, respectively. (b) Reweighted average (standard error[a]) values of angles between subdomains for each protein cluster. **O** denotes 'open', **M** denotes 'mid-open', and **C** denotes 'closed'.

the number of independent observations. We therefore also estimated the effective sample size $N_{\text{eff}} = N/\tau_{\text{int}}$ for each replica/cluster/angle, where τ_{int} is the integrated autocorrelation time (FFT autocorrelation, Geyer's initial-positive-sequence estimator[3]). Pooled N_{eff} per cluster was ∼50–100, three to four orders of magnitude below the raw frame counts, reflecting that individual 3×10^5-frame replicas typically remain within a single cluster for their full duration (Fig. S#).

To assess whether the mean angles were statistically distinct between clusters, Welch's *t*-test[4] was applied to all pairwise cluster combinations (^{P}M1 vs ^{P}M2, ^{P}M1 vs ^{P}M3, ^{P}M2 vs ^{P}M3) for each of the three inter-domain angles, as an initial, frame-level screen. Welch's *t*-test was chosen over Student's *t*-test because it does not assume equal variances between clusters, which is appropriate given the different population sizes and spread observed across the three conformational states. The test statistic is given by:

$$t = \frac{\bar{x}_1 - \bar{x}_2}{\sqrt{\dfrac{s_1^2}{n_1} + \dfrac{s_2^2}{n_2}}} \tag{2}$$

where $\bar{x}_1$ and $\bar{x}_2$ are the sample means, s_1^2 and s_2^2 are the sample variances, and n_1 and n_2 are the number of frames in each cluster.

Because this frame-level test overstates significance (see above), two autocorrelation-aware statistics were added. First, the same formula was recomputed with n_1, n_2 replaced by the pooled N_{eff}. Second, a replicate-level comparison was performed: a CE2-reweighted mean angle was computed independently for each of the 12 (condition, replica) trajectories with ≥ 200 frames in a given cluster, and the resulting sets of replicate-level means were compared between clusters via a label-shuffle permutation test with Hedges' effect size g.[5] Cluster occupancy proved strongly replica-specific in this system (individual replicas typically remained in one cluster for their full length; Fig. S#), so replicate-level means could not be paired by replica identity and were instead treated as independent samples. Where fewer than two qualifying replicates were available for a cluster (most ^{P}M3 comparisons), the N_{eff}-corrected Welch's test and effect size were reported in place of the permutation test. Effect sizes, rather than *p*-values alone, are emphasized as the primary measure of separation between clusters.

Statistical significance was defined at three levels: $p < 0.05$ (*), $p < 0.01$ (**), and $p < 0.001$ (***); ns denotes $p \geq 0.05$. Effect sizes follow conventional Hedges' g thresholds ($|g| < 0.2$ negligible, 0.2–0.5 small, 0.5–0.8 medium, > 0.8 large).[5]

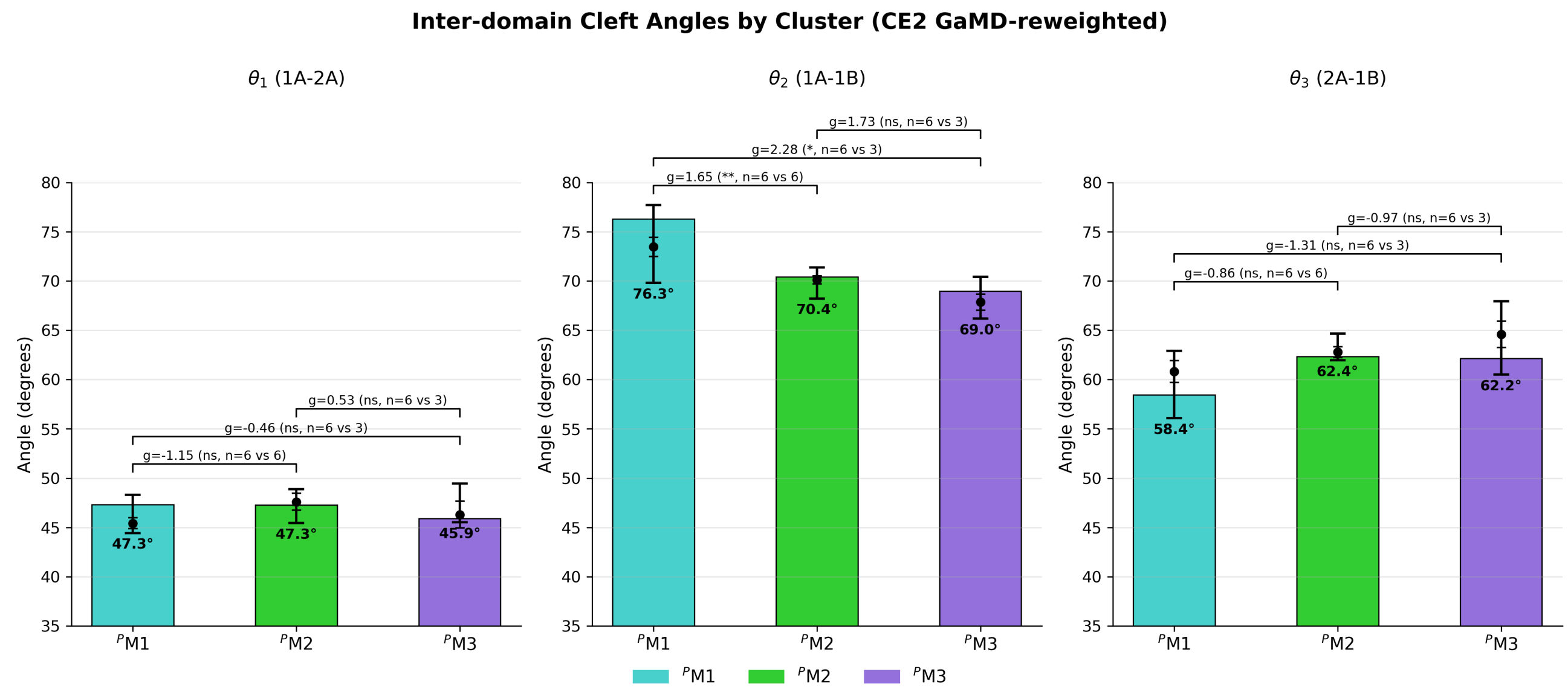

Figure S6: Reweighted mean inter-domain cleft angles for each conformational cluster with pairwise statistical significance. Bar plots show the CE2 GaMD-reweighted mean values, pooled across all four ligand conditions and replicates, of (**Left**) θ_1 (1A-2A cleft angle), (**Center**) θ_2 (1A-1B cleft angle), and (**Right**) θ_3 (2A-1B cleft angle) for clusters ^{P}M1 (medium turquoise), ^{P}M2 (lime green), and ^{P}M3 (medium purple). Error bars are the 95% block-bootstrap CI on the pooled mean; black circles with whiskers give the replicate-level mean ± SEM (see text). Bracket annotations report Hedges' g and significance from the replicate-level permutation test where ≥ 2 qualifying replicates were available per cluster, or from the N_{eff}-corrected Welch's test otherwise, together with the number of qualifying replicates (n).
Significance: $^{***}p < 0.001$, $^{**}p < 0.01$, $^{*}p < 0.05$, ns $p \geq 0.05$.

Reweighted Angle Probability Distributions

To visualize the conformational heterogeneity of each cluster with respect to the inter-domain cleft angles, reweighted probability distributions of θ_1, θ_2, and θ_3 were computed independently for each conformational cluster (^{P}M1, ^{P}M2, ^{P}M3), pooled across all conditions/replicates (Fig. S10) and, separately, within each ligand condition (Fig. S#). Distributions were computed using the same adaptive quantile-binned CE2 reweighting described above (rather than a fixed 50-bin histogram with direct exponential weights), giving a reweighted population $\tilde{P}_k$ for each angle bin:

$$P(\theta_k) = \sum_{i \in \mathrm{bin}_k} w_i \tag{3}$$

The reweighted mean angle $\langle \theta \rangle$ for each cluster (the CE2 population-weighted average, as above) is indicated in the distribution plots by a vertical dashed line, with a shaded 95% block-bootstrap CI band and the effective sample size N_{eff} reported in the legend. The width of each distribution reflects the conformational flexibility of the corresponding cluster along that inter-domain degree of freedom. Narrow distributions indicate conformationally rigid states, while broad or multimodal distributions indicate greater flexibility or the presence of multiple sub-states within a cluster; given the replica-specific occupancy noted above,

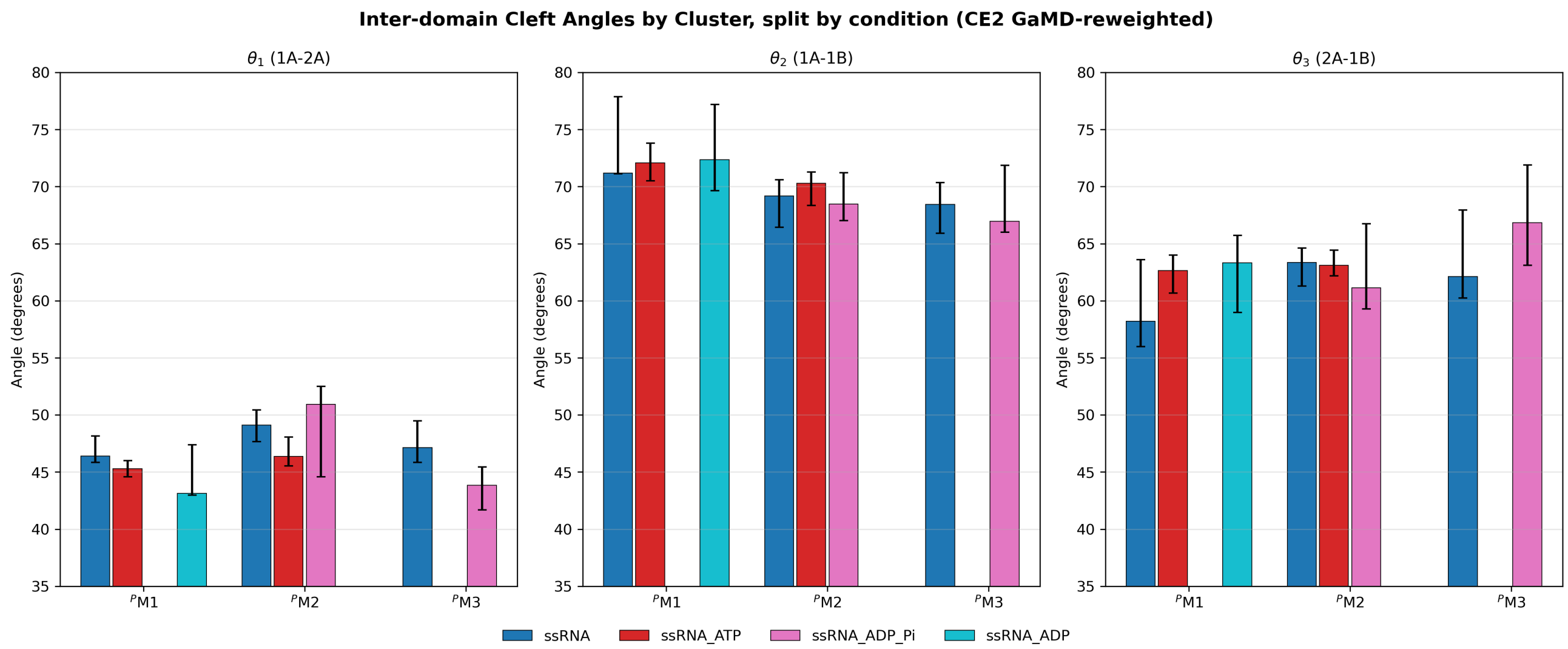

Figure S7: Reweighted mean inter-domain cleft angles by cluster, broken out by ligand condition (apo ssRNA, +ATP, +ADP+P_i, +ADP), computed as in Fig. S6 using only the frames from each condition. A cluster/condition combination is omitted where that cluster was not substantially populated ($<$ 200 frames) under that condition, consistent with the replica-specific occupancy discussed in the text.

pooled distributions here can also reflect a small number of distinct, narrow replica sub-populations rather than continuous sampling of one basin (see the by-condition breakdown, Figure S9).

The probability distributions for all three clusters were overlaid on the same panel for each angle to facilitate direct comparison of their positions and widths. Clusters ^{P}M1, ^{P}M2, and ^{P}M3 are represented in medium turquoise, lime green, and medium purple, respectively.

Per-residue Linear Discriminant Contribution Analysis

To identify the specific residues driving the conformational differences between the three protein clusters (^{P}M1, ^{P}M2, ^{P}M3), the linear discriminant (LD) vectors obtained from LDA were decomposed into per-residue contributions. The LD vectors, of dimension $N_{\text{atoms}} \times 3$, where $N_{\text{atoms}} = 446$ C_α atoms (residues 151–596), were reshaped into a three-dimensional array of shape $(N_{\text{atoms}}, 3, N_{\text{LDs}})$, where the second dimension corresponds to the Cartesian components (x, y, z) of each residue's contribution to each LD vector. The per-residue magnitude of contribution to each LD vector was then computed as the Euclidean norm of the Cartesian components:

$$m_{i,k} = \sqrt{x_{i,k}^2 + y_{i,k}^2 + z_{i,k}^2} \tag{4}$$

where $m_{i,k}$ is the magnitude of residue i's contribution to the k-th linear discriminant, and $x_{i,k}$, $y_{i,k}$, $z_{i,k}$ are the corresponding Cartesian components of the LD vector for residue i.

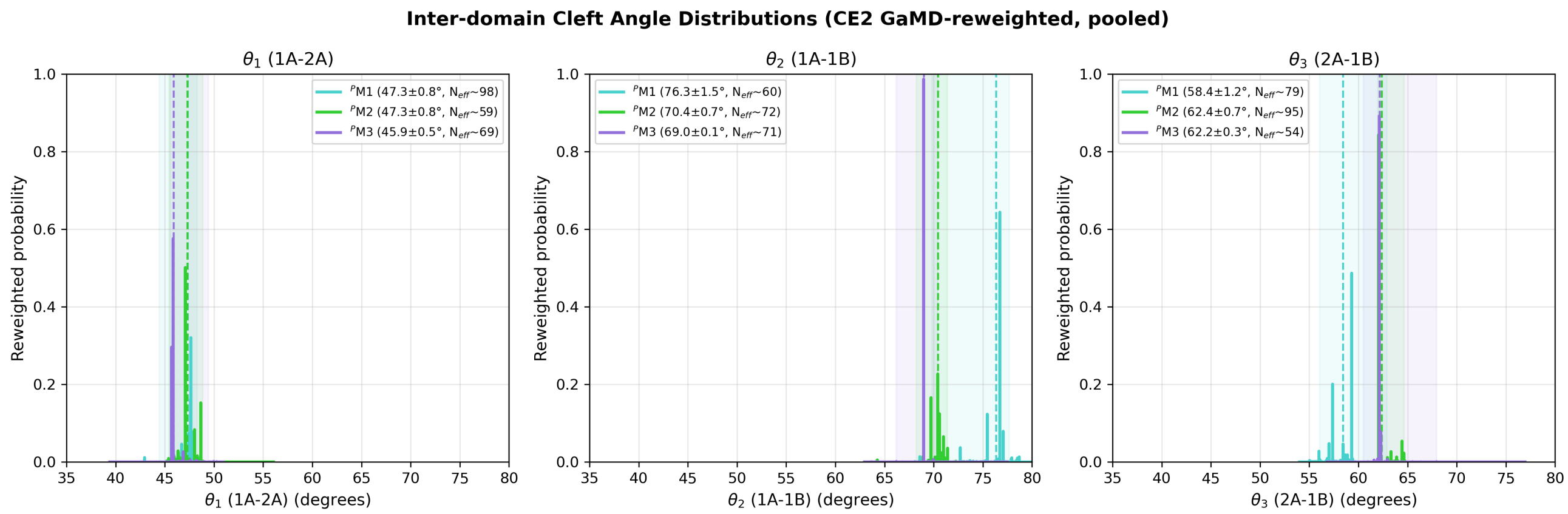

Figure S8: Reweighted probability distributions of inter-domain cleft angles for each conformational cluster, pooled across all conditions/replicates

. (**Left**) θ_1 (1A-2A cleft angle), (**Center**) θ_2 (1A-1B cleft angle), and (**Right**) θ_3 (2A-1B cleft angle) for clusters ^{P}M1 (medium turquoise), ^{P}M2 (lime green), and ^{P}M3 (medium purple), computed via adaptive quantile-binned CE2 reweighting, normalized to unit probability. Vertical dashed lines indicate the reweighted mean angle, shaded bands its 95% block-bootstrap CI, and N_{eff} (legend) the autocorrelation-corrected effective sample size. Narrow distributions reflect conformationally rigid states, while broad or multimodal distributions indicate greater flexibility or multiple sub-states within a cluster.

To enable direct comparison of contributions across residues and between LD vectors, the per-residue magnitudes were normalized to the interval $[0, 1]$ using min-max normalization:

$$\tilde{m}_{i,k} = \frac{m_{i,k} - \min_j(m_{j,k})}{\max_j(m_{j,k}) - \min_j(m_{j,k})} \tag{5}$$

where $\tilde{m}_{i,k}$ is the normalized magnitude of residue i along the k-th LD vector, and the minimum and maximum are taken over all N_{atoms} residues. Residues with $\tilde{m}_{i,k}$ approaching unity are the primary structural determinants distinguishing the three conformational states along that LD axis, while residues with $\tilde{m}_{i,k}$ approaching zero remain relatively invariant across clusters.

The normalized per-residue LD magnitudes were visualized as bar plots as a function of residue index for LD1 and LD2 separately, with bars colored on a blue-to-red scale (coolwarm colormap) corresponding to low-to-high normalized magnitude. The top 10 residues with the highest normalized LD magnitude were identified and labeled for each LD vector to highlight the most discriminating regions of the protein. Additionally, the normalized magnitudes were mapped onto the representative structure of each conformational cluster as crystallographic B-factors to produce structure figures highlighting the spatial distribution of conformationally discriminating residues.

Comparison of Mutant and Wild-type Conformational Ensembles

To assess whether the L405D mutant samples conformational states accessible to the wild-type enzyme, we compared the reweighted probability distributions of both trajectories projected onto the linear discriminant (LD) axes derived independently from each system. Mutant and wild-type C_α trajectories (residues 151–596) were flattened into feature vectors of dimension $N_{\text{atoms}} \times 3$ and projected onto the first two LD vectors derived from the mutant LDA model, as well as onto the first LD vector derived from the wild-type LDA model, yielding one-dimensional conformational projections for each system on each axis. Frame weights were computed independently for each trajectory using the exponential reweighting scheme described above.

The similarity between the mutant and wild-type reweighted distributions was quantified using two complementary metrics. First, the Jensen–Shannon (JS) divergence was computed as:

$$JS(P\|Q) = \frac{1}{2}D_{\text{KL}}(P\|M) + \frac{1}{2}D_{\text{KL}}(Q\|M) \tag{6}$$

where P and Q are the reweighted probability distributions of the mutant and wild-type projections, respectively, $M = \frac{1}{2}(P+Q)$ is their mixture distribution, and D_{KL} denotes the Kullback–Leibler divergence. Both distributions were histogrammed into 50 equally spaced bins over the combined range of the two projections, normalized to unit probability, and regularized with a small epsilon ($\epsilon = 10^{-10}$) to avoid undefined logarithms. The JS divergence ranges from 0 (identical distributions) to 1 (completely non-overlapping distributions), with values above 0.5 indicating that the two ensembles are more different than alike.

Second, the overlap coefficient (OC) was computed as:

$$OC(P, Q) = \sum_k \min(P_k, Q_k) \tag{7}$$

where the sum runs over all histogram bins k. The overlap coefficient ranges from 0 (no shared conformational space) to 1 (identical distributions) and provides a direct measure of the fraction of conformational space sampled by both systems. Both metrics were computed on the mutant-derived LD1 and LD2 axes, as well as on the wild-type-derived LD1 axis, to ensure that the observed separation was not an artifact of the coordinate system used for projection.

References

(1) Miao, Y.; Sinko, W.; Pierce, L.; Bucher, D.; Walker, R. C.; McCammon, J. A. Improved reweighting of accelerated molecular dynamics simulations for free energy calculation. *Journal of Chemical Theory and Computation* **2014**, *10*, 2677–2689.

(2) Efron, B.; Tibshirani, R. J. *An Introduction to the Bootstrap*, 1st ed.; Chapman and Hall/CRC, 1994.

(3) Geyer, C. J. Practical markov chain monte carlo. *Statistical science* **1992**, 473–483.

(4) Welch, B. L. The generalization of 'STUDENT'S' problem when several different population varlances are involved. *Biometrika* **1947**, *34*, 28–35.

(5) Hedges, L. V. Distribution theory for Glass's estimator of effect size and related estimators. *journal of Educational Statistics* **1981**, *6*, 107–128.

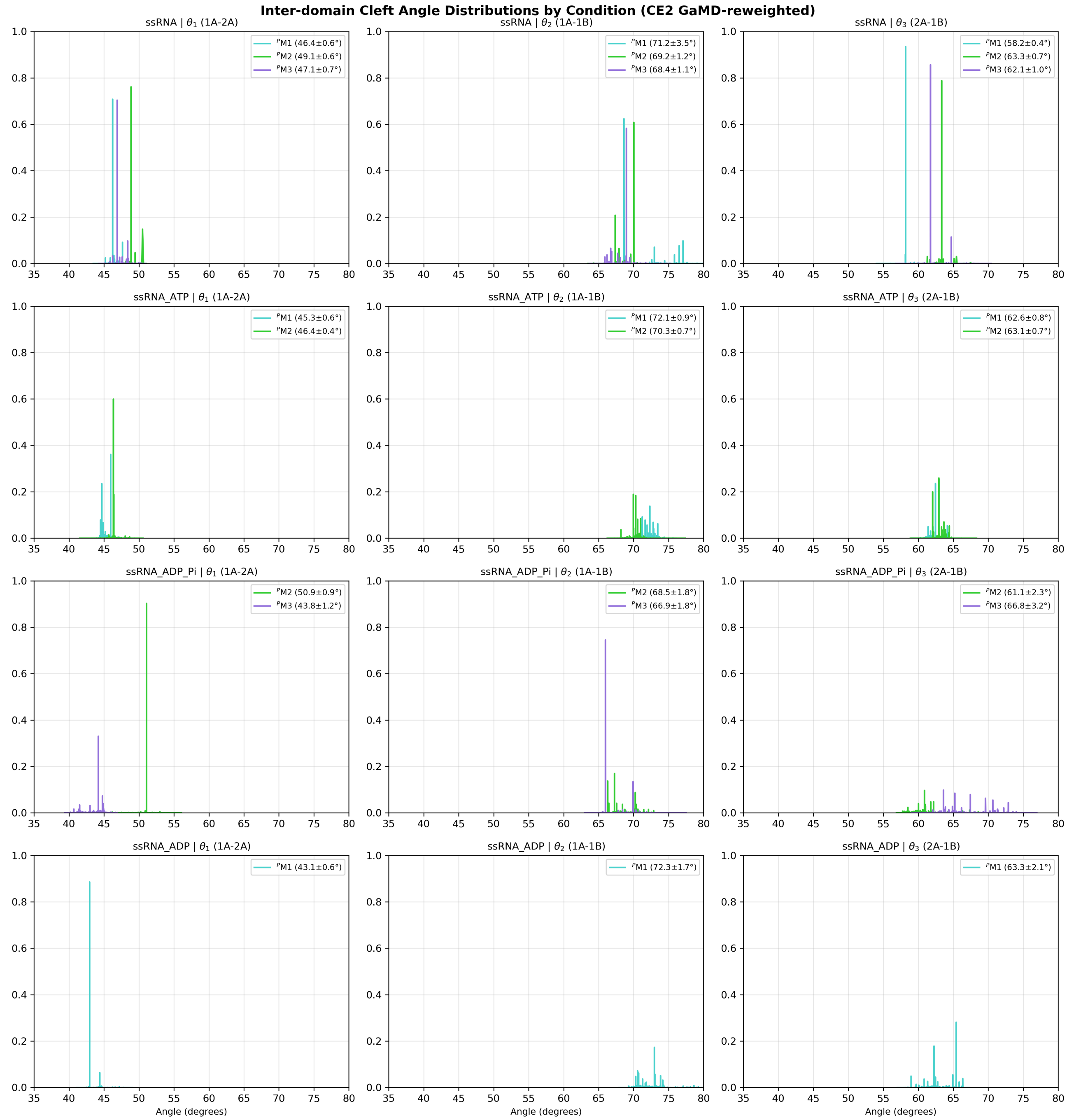

Figure S9: Reweighted probability distributions of inter-domain cleft angles by cluster, broken out by ligand condition (rows) and angle (columns), computed as in Fig. S10 using only the frames from each condition. Legend entries appear only for clusters substantially populated (≥ 200 frames) under that condition.

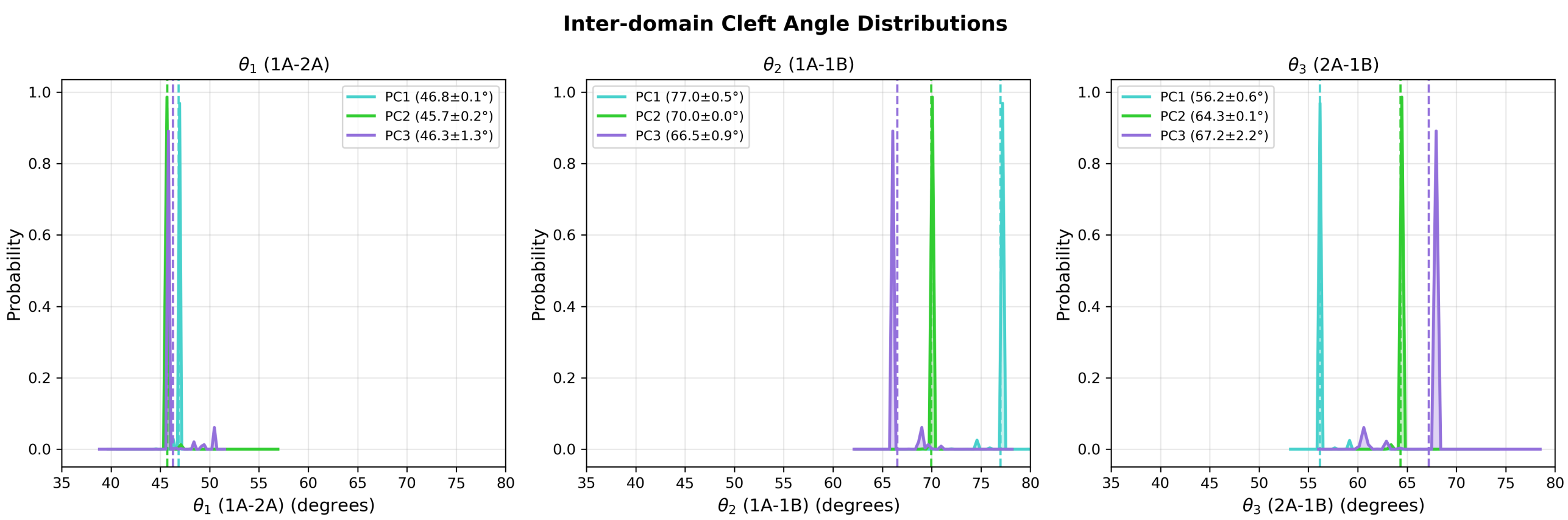

Figure S10: Reweighted probability distributions of inter-domain cleft angles for each conformational cluster. (**Left**) Distribution of θ_1 (1A-2A cleft angle), (**Center**) distribution of θ_2 (1A-1B cleft angle), and (**Right**) distribution of θ_3 (2A-1B cleft angle) for clusters ^{P}M1 (medium turquoise), ^{P}M2 (lime green), and ^{P}M3 (medium purple). Distributions were computed by histogramming reweighted frame contributions into 50 equally spaced bins and normalizing to unit probability. Vertical dashed lines indicate the reweighted mean angle for each cluster. Narrow distributions reflect conformationally rigid states, while broad or multimodal distributions indicate greater flexibility or the presence of multiple sub-states within a cluster. Frame weights were derived from the bias potential using exponential reweighting at $T = 300$ K (see Methods).

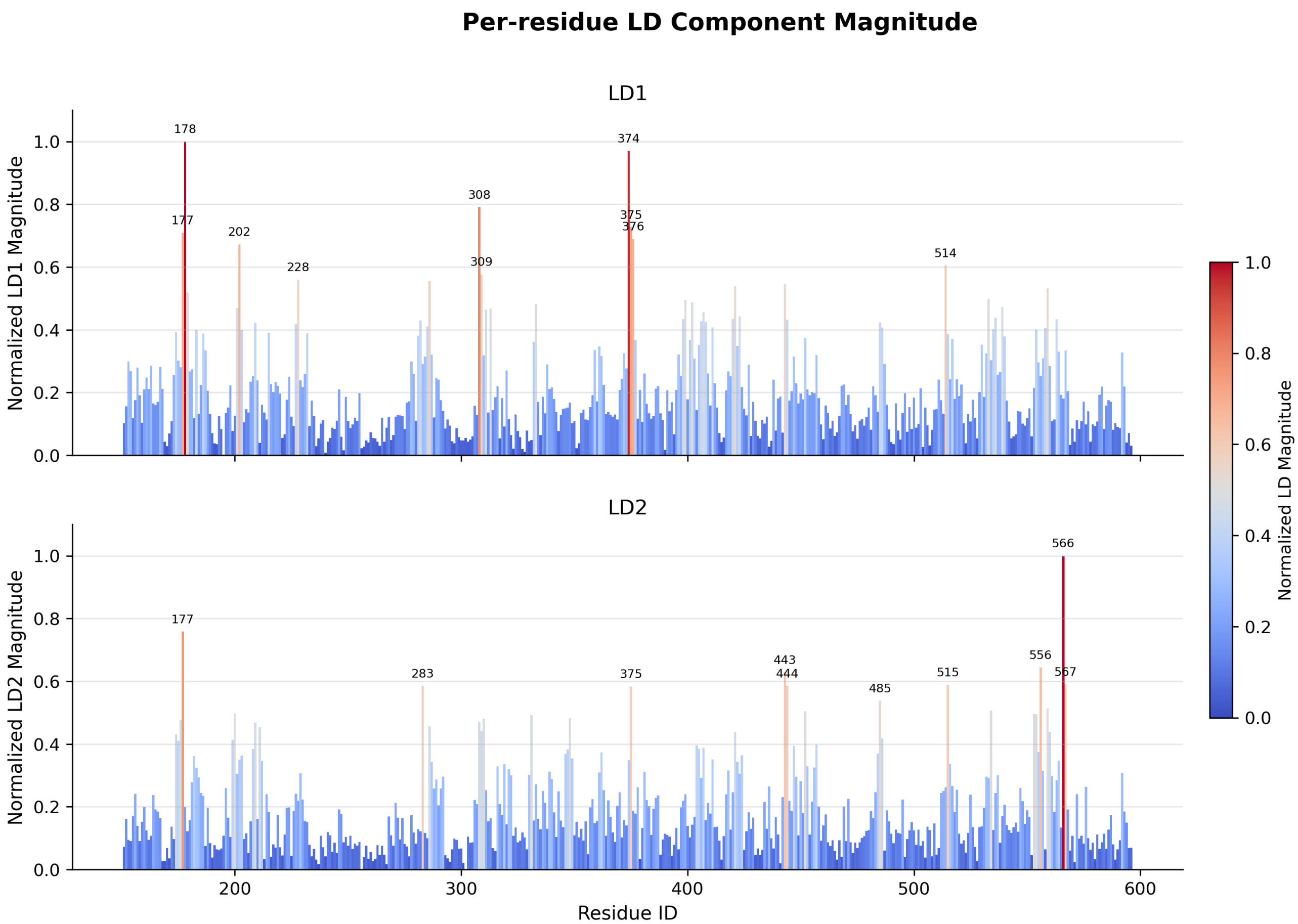

Figure S11: Per-residue contributions to the linear discriminant (LD) vectors separating the three conformational clusters. (**Top**) Normalized LD1 magnitude and (**Bottom**) normalized LD2 magnitude as a function of residue index (residues 151–596). The per-residue magnitude was computed as the Euclidean norm of the (x, y, z) components of each residue's contribution to the LD vector, $\sqrt{x^2 + y^2 + z^2}$, and normalized to $[0, 1]$. Residues with high normalized magnitude (approaching 1, red) are the primary structural determinants distinguishing the three conformational states, while residues with low magnitude (approaching 0, blue) remain relatively invariant across clusters. The top 10 contributing residues are labeled for each LD vector.

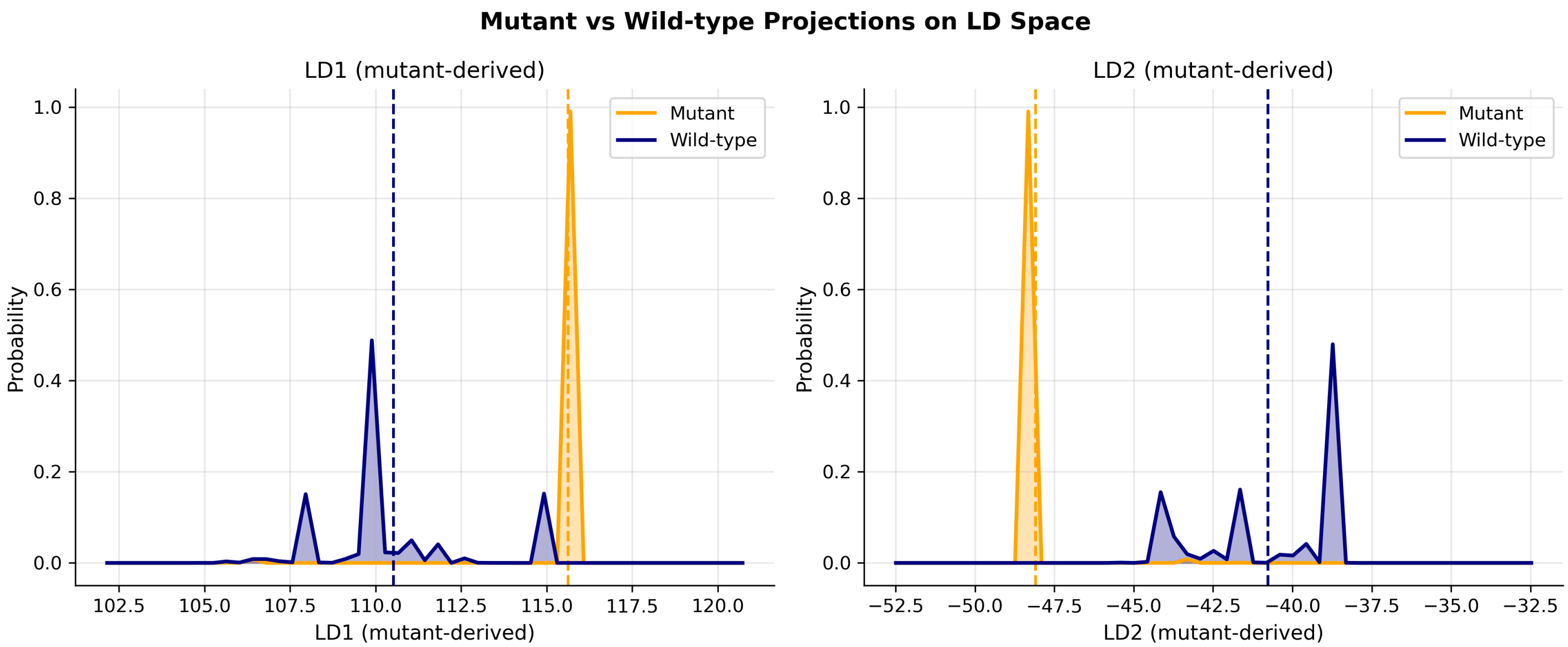

Figure S12: Comparison of mutant and wild-type conformational ensembles projected onto the mutant-derived linear discriminant axes. Reweighted probability distributions of the L405D mutant (orange) and wild-type (navy) trajectories projected onto (**Left**) LD1 and (**Right**) LD2, both derived from the mutant LDA model. Vertical dashed lines indicate the reweighted mean projection value for each system. The mutant distribution is narrow and highly localized, while the wild-type distribution is broader and multimodal, sampling multiple distinct regions along each axis. The near-complete absence of distributional overlap ($OC < 0.002$ for both axes) and large Jensen–Shannon divergence ($JS > 0.69$) confirm that the two systems occupy largely distinct regions of conformational space, indicating that L405D stabilizes conformations that are rarely, if at all, accessible to the wild-type enzyme.
